# *Cripto-1* as a novel therapeutic target for triple negative breast cancer

**DOI:** 10.18632/oncotarget.4182

**Published:** 2014-05-19

**Authors:** Nadia P. Castro, Natalie D. Fedorova-Abrams, Anand S. Merchant, Maria Cristina Rangel, Tadahiro Nagaoka, Hideaki Karasawa, Malgorzata Klauzinska, Stephen M. Hewitt, Kajal Biswas, Shyam K. Sharan, David S. Salomon

**Affiliations:** ^1^ Tumor Growth Factor Section, Mouse Cancer Genetics Program, National Cancer Institute, Frederick, MD, USA; ^2^ CCRIFX Bioinformatics Core, National Cancer Institute, Bethesda, MD, USA; ^3^ Laboratory of Pathology, Center for Cancer Research, National Cancer Institute, Bethesda, MD, USA; ^4^ Genetics of Cancer Susceptibility Section, Mouse Cancer Genetics Program, National Cancer Institute, Frederick, MD, USA

**Keywords:** cripto-1, notch4, epithelial-mesenchymal plasticity, mouse model, triple-negative breast cancer

## Abstract

Triple-negative breast cancer (TNBC) presents the poorest prognosis among the breast cancer subtypes and no current standard therapy. Here, we performed an in-depth molecular analysis of a mouse model that establishes spontaneous lung metastasis from JygMC(A) cells. These primary tumors resembled the triple-negative breast cancer (TNBC) both phenotypically and molecularly. Morphologically, primary tumors presented both epithelial and spindle-like cells but displayed only adenocarcinoma-like features in lung parenchyma. The use of laser-capture microdissection combined with Nanostring mRNA and microRNA analysis revealed overexpression of either epithelial and miRNA-200 family or mesenchymal markers in adenocarcinoma and mesenchymal regions, respectively. *Cripto-1*, an embryonic stem cell marker, was present in spindle-like areas and its promoter showed activity in primary tumors. *Cripto-1* knockout by the CRISPR-Cas9 system inhibited tumor growth and pulmonary metastasis. Our findings show characterization of a novel mouse model that mimics the TNBC and reveal *Cripto-1* as a TNBC target hence may offer alternative treatment strategies for TNBC.

## INTRODUCTION

Among all cancer types, breast cancer (BC) has the highest incidence and is the second most common cause of cancer death in women in the United States [1]. The most lethal aspect of BC is its capacity to metastasize to the lungs, brain, liver and bones. Within the molecular subtypes, basal-like or triple-negative breast cancers (TNBC), which lack expression of estrogen receptor (ER), progesterone receptor (PR) and HER2/neu tyrosine kinase receptor, are generally associated with the worst prognosis due to its high resistance to chemotherapy [2, 3]. The poor clinical prognosis, lack of curative therapies and metastasis demand an imperative identification of effective molecular targets and related therapies in TNBC.

The metastatic process has several steps that are related to normal embryonic development. For instance, the epithelial-mesenchymal transition (EMT) is critical during normal embryonic development, fibrosis and wound healing, but also contributes to tumor invasion and metastasis [4]. Experimental evidence indicates that interconversion between EMT and the reverse process of mesenchymal-epithelial transition (MET) can occur during embryogenesis and fibrosis as well as during early stages of metastatic progression in colorectal cancer and BC, particularly at the invasive front of primary tumors and in metastatic sites, respectively [4].

Cancer stem cells (CSC), also known as tumor-initiating cells, share several characteristics associated with normal tissue stem cells and have been identified in human tumors as possessing long-term self-renewal potential, quiescent properties and resistance to chemotherapy and radiotherapy [5]. CSC were first identified in hematopoietic system malignancies and further characterized in solid tumors of the breast, lung, prostate, colon, brain, head and neck, and pancreas [6]. Interestingly, a gain in stem-like features in tumors has been associated with mesenchymal/EMT-like state and a more aggressive tumor phenotype [7]. Recent studies have shown that TNBC is highly enriched for EMT and CSC markers [8, 9]. Additionally, the Wnt/β-catenin, Notch, TGF-β and Hedgehog pathways contribute to the self-renewal of stem cell/progenitor populations and the initiation of EMT during embryonic development and in differentiated adult tissues, such as the skin, colon and mammary glands [6], and are also hyperactivated during tumorigenesis [10].

Cripto-1, a TGF-β family member is critically important in early embryogenesis, stem cell maintenance and malignant progression [6]. Cripto-1, also known as Tdgf-1, is an oncofetal, GPI-anchored/secreted signaling protein that is involved in regulating the formation of the primitive streak, specification of mesoderm and endoderm during gastrulation, and establishment of left/right asymmetry of developing organs [6]. Also, Cripto-1 has been shown to play a role during EMT and as a stem cell regulator [6, 11]. Notably, recent reports have demonstrated a physical interaction between Cripto-1 and all four Notch receptors suggesting that Cripto-1 facilitates the posttranslational maturation of Notch receptors [6, 12]. The Notch signaling pathway is also critically important for normal development in several tissues [13]. Notch proteins include 4 transmembrane receptors (NOTCH1-4). In particular, NOTCH4 has been shown to be relevant in the maintenance of a human BC stem cell population [14]. More recently, a few mutations have been observed in the Notch1/2 receptors in TNBC [15].

In this study, we extensively characterized a clinically relevant mouse model, which phenotypically and at the gene-expression level resembles the human TNBC molecular subtype. In this model, we used the JygMC(A) cell line isolated from a spontaneous mammary carcinoma, which arose in a Chinese wild mouse (Mus musculus Sub-Jyg) [16, 17]. It spontaneously metastasizes to several organs, including the lungs and liver when inoculated subcutaneously into nude mice [17]. The JygMC(A) cells contain an insertion of the mouse mammary tumor virus (MMTV) in the Int3/Notch4 locus suggesting that MMTV-induced activation of Int3 is manifested in the absence of the regulatory action of the extracellular domain (ligand-independent), yet requires gamma-secretase enzymatic processing for full activation of the pathway [18, 19]. This animal model is uniquely capable of spontaneous epithelial-mesenchymal plasticity that exhibits lung metastasis when injected into the mammary fat pad. To identify a TNBC target, we assessed the expression of genes associated with EMT-MET and the maintenance of embryonic stem cells (ESC) using targeted and global gene expression array and microRNA expression in primary mammary tumors and pulmonary metastases. We found that this metastatic model resembles the human TNBC subtype, revealing Cripto-1 as a novel therapeutic target for TNBC.

## RESULTS

### Orthotopic metastasis of JygMC(A) cells

**Figure 1:**
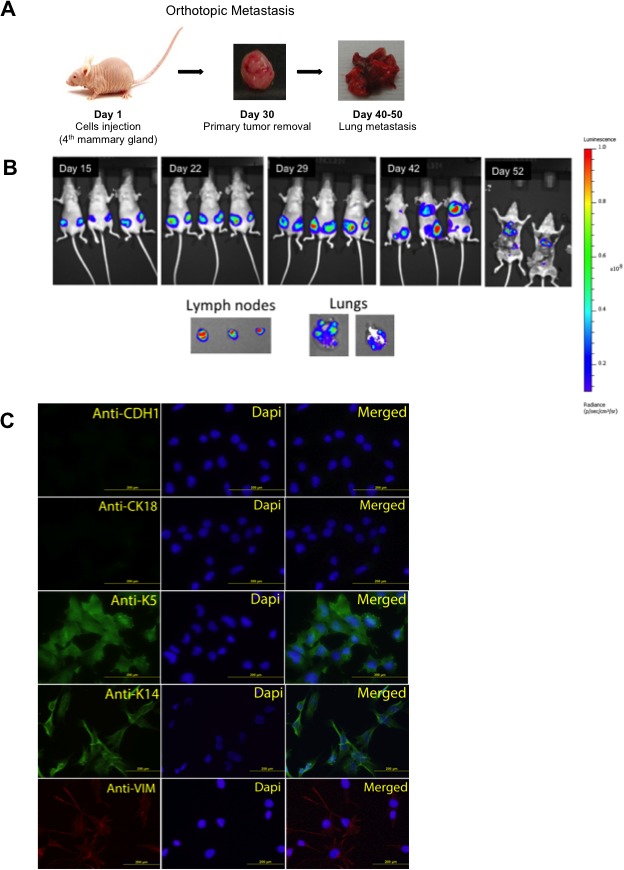
Orthotopic metastasis of JygMC(A) cells and epithelial mesenchymal characterization *in vitro* Lung metastasis by orthotopic cell injection in Balb/C nude mice. **B.** Representation of bioluminescent imaging of animals. Animals imaged at day 15, 22, 29, 42 and 52 post JygMC(A)-GFP/Luc cell injection. Ex-vivo imaging of lymph nodes and lung metastases. **C.** Immunofluorescence of luminal epithelial and basal mesenchymal markers in JygMC(A) parental cells. Epithelial marker: CDH1 (Alexa 488), luminal marker: CK18 (Alexa 488), basal marker: K5 and K14 (Alexa 488) and mesenchymal marker: Vimentin (Alexa 594). Nuclear staining in blue (DAPI). Scale bars: 200 μm.

In order to assess the metastatic progression of JygMC(A) mouse mammary tumor cells, we generated JygMC(A) cells stably expressing the firefly luciferase and eGFP reporter genes (JygMC(A)-GFP/Luc) to facilitate the *in vivo* tracking of primary and metastatic tumor cells. Cells were injected into the fourth mammary fat pad on day 1, after 30 days primary tumors were removed. Lung metastases were accessed on day 40-50 (see schematic chart of orthotopic metastasis on Figure 1A). Animals were imaged in different time-points using *in vivo* bioluminescent imaging (Figure 1B). To determine whether the tagged cells would have similar metastatic and tumor-initiating capacity as the parental cells, four groups of 3 animals were injected as follows: Group A = 500,000 cells, Group B = 50,000 cells, Group C = 5,000 cells, Group D = 500 cells. At day 30 after injection, the primary tumors were removed, except for Group D where tumors were removed at day 52. The parental and tagged cells exhibited similar tumor volumes (Figure S1A), primary tumor incidence, tumor-initiating capacity, metastases frequencies (Table S1). JygMC(A) cells exhibited high propensity to metastasize to the lungs and liver, and, to a small extent, the spleen and kidney when injected into the fourth mammary fat pad, as shown in Table S1.

### JygMC(A) cells exhibit a basal-mesenchymal phenotype and primary mammary tumors resemble the human TNBC by immunohistochemistry

In order to phenotypically characterize the JygMC(A) cells, we performed immunofluorescence assays using luminal (CK18), epithelial (CDH1), basal (K5 and K14) and mesenchymal (Vimentin) markers (Figure 1C). The results showed that the JygMC(A) cells in culture exhibit a basal-like mesechymal phenotype.

**Figure 2:**
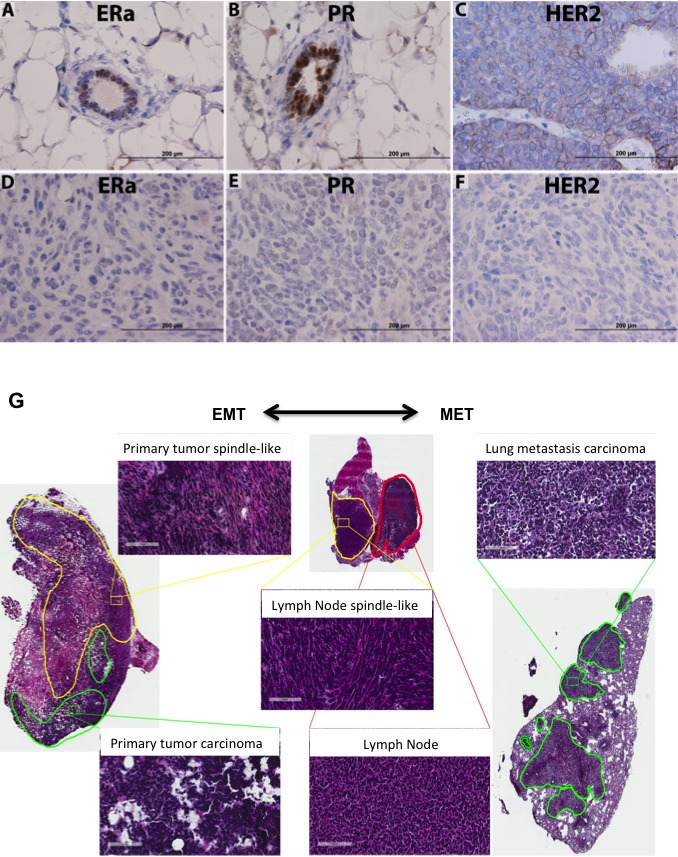
JygMC(A) mammary primary tumor molecular phenotype, histology and EMT-MET plasticity Analysis of normal adjacent tissue by immunohistochemistry of ERα **A.** PR **B.** and HER2/neu staining of a wild-type MMTV-Neu/Her2 tumor **C.** Analysis of primary mammary tumor tissue of ERα **D.** PR **E.** and HER2/neu **F.** Positive antibody signals are shown in brown, and the hematoxylin counterstain is shown in blue. Scale bars: 200μm. **G.** Histological tumor classification. Primary tumor possesses two types of cells: undifferentiated adenocarcinoma type (green line) and areas composed of atypical spindle-shaped cells suggesting EMT (yellow line). Lung metastasis morphology typical of the epithelial primary adenocarcinoma suggesting MET is present in the lung parenchyma. Lymph node tissue (red line) showing areas of atypical spindle-shaped cells (yellow line). Shown are hematoxylin and eosin staining. Scale bars: 100 μm.

Immunohistochemistry on histological samples determined that JygMC(A) primary tumors resembled the human TNBC molecular subtype. Surrounding normal mammary tissue that was adjacent to primary tumors showed positive nuclear staining for estrogen receptor alpha (ERα) and progesterone receptor (PR) (Figure 2A and 2B). Transgenic MMTV-Neu/HER2 mammary tumors show HER2 expression (Figure 2C). The lack of ERα, PR and HER2 expression indicates that the JygMC(A) primary mammary tumors share a human TNBC phenotype (Figure 2D, 2E and 2F, respectively).

### JygMC(A) primary mammary tumors exhibit epithelial-mesenchymal plasticity

Recent evidence suggests that epithelial-mesenchymal plasticity may be crucial in the metastatic process [20]. In this study, we demonstrated that JygMC(A) mammary tumors histologically exhibit two types of cell morphology: (i) an undifferentiated adenocarcinoma type and (ii) atypical spindle-shaped mesenchymal-like cells which are suggestive of EMT occurring in these primary lesions. Notably, only the adenocarcinoma phenotype was observed in the metastases found in the lung parenchyma, suggesting that epithelial tumor cells may have had undergone EMT in primary tumors followed by MET in the lung parenchyma (Figure 2G). The atypical spindle-shaped mesenchymal-like cells were observed in the axillary mammary lymph nodes (Figure 2G). Taken together these results suggested that a series of EMT-MET transitions might occur during the metastatic process in this mouse model.

### Targeted mRNA gene expression profiling of JygMC(A) primary mammary tumors and lung metastases identifies ESC and EMT-MET Markers

**Figure 3:**
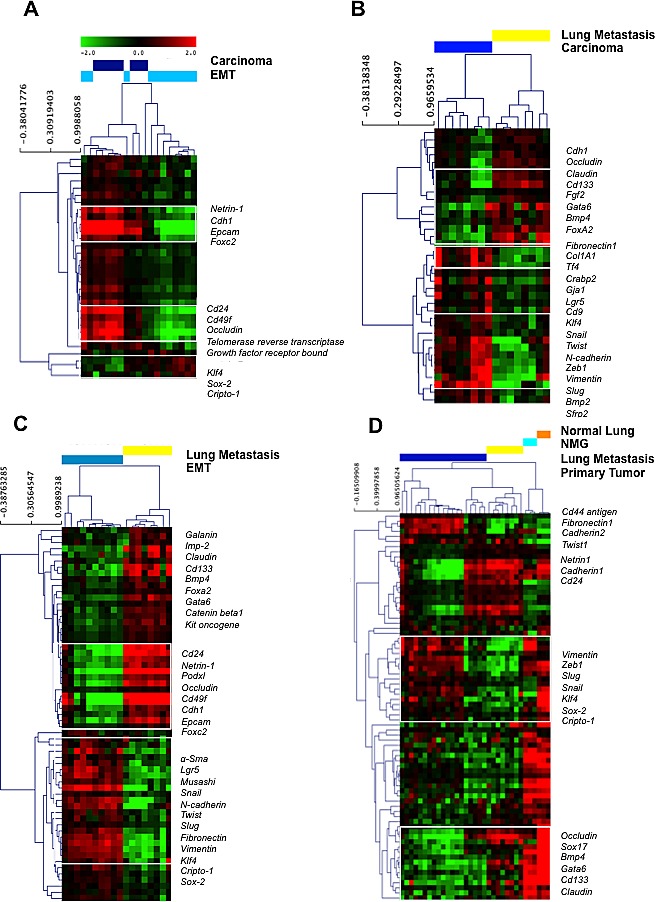
Gene expression profiles of laser capture microdissected tumor progression samples through unsupervised hierarchical clustering Primary tumor adenocarcinoma versus EMT-like areas; **B.** Primary tumor adenocarcinoma versus lung metastasis; **C.** Primary tumor EMT-like areas versus lung metastasis; **D.** Primary tumor, lung metastasis, normal mammary gland and normal lung parenchyma. Each row represents a single gene and each column a single sample. The red color indicates up-regulation, the green color indicates down-regulation, and the black color indicates no change in expression level compared with the reference sample. The gray color indicates that no intensity was detected. EMT: epithelial-to-mesenchymal transition.

To examine the gene expression profile of these mixed cell populations in the primary tumors and lung metastases, we integrated the use of laser capture microssection (LCM) and NanoString Technologies to identify expression levels of ESC and EMT-MET markers (Dataset S1A). Since ESCs are known for their ability to differentiate into somatic tissues derived from different germ layer cell types (ectoderm, mesoderm and endoderm) [7], we reasoned that the re-expression of ESC genes might also play a role during this metastatic process. Based on a collection of published literature, we built a customized NanoString nCounter^®^ Gene Expression Codeset that contained significant ECS and EMT-MET markers, and targeted gene expression profiling on the following samples: primary tumor adenocarcinoma and EMT-like, lung metastases, normal mammary gland (NMG) and normal lung parenchyma. Figure 3 shows the most notably altered genes between these sample groups. An examination of gene expression patterns of the primary tumor that contained mixed areas revealed 31 genes differentially expressed (Figure 3A). Overexpression of ESC markers segregated distinctly in the epithelial cell-populated regions (Cd24 and Cd49f) and spindle-like cell areas (Klf4, Sox2 and Cripto-1). Moreover, overexpression of epithelial markers (Cadherin1, Epcam and Occludin) was observed in adenocarcinoma regions. For complete gene lists, see Dataset S1B.

We then compared gene expression in adenocarcinoma areas of primary tumor versus lung metastasis. Thirty-eight genes were identified segregating 100% of sample groups in the cluster. Even though both areas exhibited an epithelial morphology, we observed a rather distinct gene expression profile with mesenchymal molecular signature (Fibronectin, Snail, Cadherin2, Zeb1, Vimentin and Slug) in primary tumors and epithelial molecular signature (Cadherin 1, Occludin, Claudin, Cd133, Fgf2, Gata6, Bmp4) in lung metastasis (Figure 3B and Dataset S1C). Comparison between EMT-like areas in primary tumors with epithelial-like areas in lung metastases generated a list of 65 genes that segregated 100% of sample groups in the cluster. We observed overexpression of several EMT markers (α-Sma, Snail, Cadherin 2, Twist, Slug, Fibronectin, Vimentin, Zeb1) and ESC markers (Klf4, Sox2, Cripto-1) in the EMT-like areas but observed overexpression of MET markers (Occludin, Cadherin1, Podxl and Epcam) in lung metastases (Figure 3C and Dataset S1D). Finally, we conducted an ANOVA test, which identified 88 genes differentially expressed among all samples with a distinct gene expression pattern in tumor samples when compared with normal tissue (Figure 3D and Dataset S1E). A group of genes overexpressed in normal lung parenchyma were underexpressed in lung metastasis (Klf4, Sox2, Cripto-1, Vimentin, Zeb1, Slug and Snail) and genes overexpressed in NMG were under expressed in primary tumors (Sox17, Bmp4, Gata6, Cd133, Occludin and Claudin). These analyses identified the ESC and EMT-MET molecular profile of JygMC(A) mammary tumors and lung metastases.

### Global gene expression profiling of JygMC(A) primary mammary tumors and pulmonary metastasis

**Figure 4:**
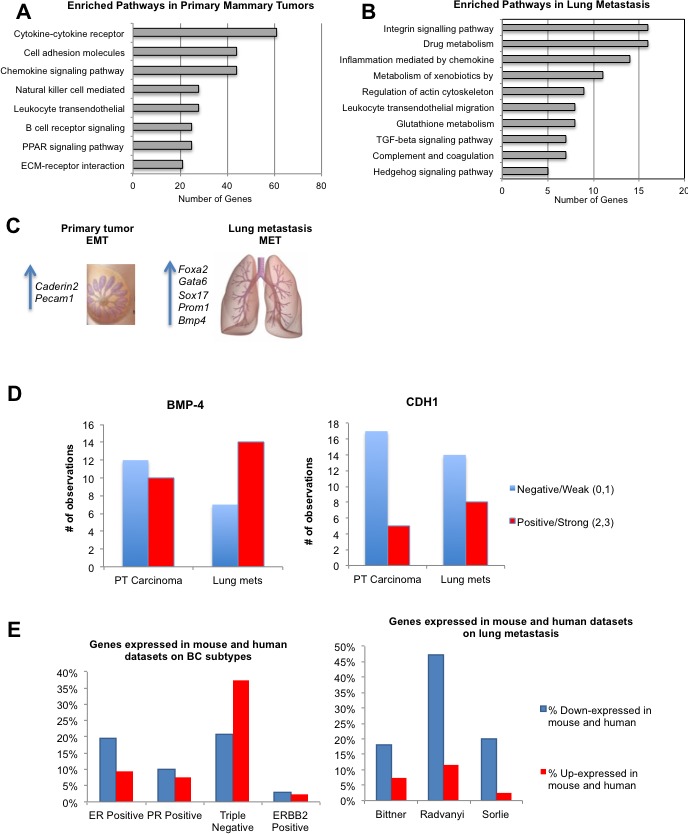
Relevant pathways, gene and protein expression in primary tumors and lung metastasis and similarities with human breast cancer Enriched pathways in primary mammary tumors. **B.** Enriched pathways in lung metastasis. **C.** Common seven genes between Nanostring and microarray analysis **D.** BMP4 and CDH1 protein expression. **E.** Differentially expressed genes between JygMC(A) primary mammary tumors and/or lung metastasis and human breast cancer subtypes and/or metastasis datasets.

To fully characterize gene expression patterns of the JygMC(A) model on a global scale, we performed whole-genome transcriptome microarray profiling of primary mammary tumors, lung metastases, NMG and normal lung parenchyma. Differential expression analysis (FDR adjusted *p*-value <0.05 and fold change (FC)>= 2) identified 2,915 genes between primary tumors and NMG including 1,089 up-regulated and 1,826 down-regulated genes (Dataset S2A). Additionally, we identified 472 genes differentially expressed between lung metastases and primary tumors including 430 up-regulated and 42 down-regulated genes (Dataset S2B). A four-way Venn diagram shows increases and decreases of expressed genes for both comparisons (Figure S1B). Using the top 1,000 differentially expressed genes (FDR adjusted *p*-value <0.05 and FC>=3), we identified eight pathways enriched in primary tumors as compared to NMG samples, such as cytokine-cytokine receptor and cell adhesion molecules (Figure 4A), and ten pathways enriched in the lung metastases as compared to the primary tumors, such as the integrin signaling pathway, chemokine-mediated inflammation and the cytokine signaling pathway (Figure 4B). A complete list of the pathways and genes involved are summarized in Dataset S2C and S2D, respectively.

To compare targeted (NanoString) and genome-wide (Affymetrix) gene expression data, we performed an unsupervised hierarchical clustering of the Affymetrix gene expression data using 98 genes (103 minus 5 controls) from the targeted gene list (Dataset S3A). The heat map in Figure S1C shows similar clustering patterns for some genes in both data sets (Figure 3D and Figure S1C). We then determined the common genes that were concordantly changed between primary tumor and lung metastases in both data sets. Seven such genes were identified (Dataset S3B). Overexpression of Cadherin2 (CDH2) and Pecam-1 was observed in the primary tumors whereas overexpression of the Foxa2, Gata6, Sox17, Prom1 and Bmp4 were detected in the pulmonary metastases (Figure 4C) (Additional qRT-PCR validation is shown in Figure S2A-F).

### BMP-4 and CDH1 protein expression is higher in lung metastases

As a validation of the targets identified in the transcriptome profiling of different tumor regions, we constructed a tissue microarray (TMA) containing normal tissue samples from 2 animals as well as mixed populations of primary tumor (adenocarcinoma and EMT-like) and lung metastases from six animals that were obtained from 3-5 different tissue cores per sample. Since a switch from Cadherin1 (CDH1) to CDH2 expression indicates EMT [21] and BMP signaling suppresses traits associated with CSC and limits BC cell proliferation [22], we verified protein expression in our model by immunohistochemical analysis. Based on the staining intensity, BMP-4 and CDH1 demonstrated a higher protein expression and/or increased positivity in lung metastases when compared with the primary tumor tissues (Figure 4D). These results were concordant with the gene expression findings. A staining representation pattern can be found in Figure S3G.

### Similarity between JygMC(A) mouse mammary tumors and human TNBC subtypes at the gene expression level

To assess the potential clinical relevance of the JygMC(A) mouse mammary tumor model and to determine whether the model resembles a specific human molecular BC subtype(s) at the mRNA level, we performed a meta-analysis using the Oncomine cancer microarray database [23]. First, we wanted to examine expression patterns of human orthologs of the 1,055 mouse genes that were differentially expressed in the JygMC(A) primary tumors versus NMG (FDR adjusted *p*-value <0.05 and FC>=3). Our comparison was limited to the mouse genes (299 up-regulated and 756 down-regulated) that had human orthologs and were represented on the arrays. To minimize the number of spurious hits, we limited our search to human studies that included gene expression (mRNA) analysis of molecular subtypes of invasive BC and had at least 150 samples (see Methods for details). Using these criteria, we identified four published human BC studies [24-27], and The Cancer Genome Atlas (TCGA) (unpublished) in Oncomine.

The most conspicuous finding from this comparison was the concordance between the expression patterns in JygMC(A) primary mouse mammary tumors and the human TNBC subtype, which is defined as ER, PR and HER2 negative. The similarity was strongest between our data and the TCGA dataset, which is the largest (593 samples) and most comprehensive (20,423 measured genes) dataset in Oncomine (Figure 4E and Dataset S4A). Thus, out of 299 genes overexpressed in the JygMC(A) primary tumors, 112, or 37%, were also significantly expressed in the TNBC subtype as compared to other subtypes in the TCGA study. One of the top-ranked genes in both datasets was FOXC1 (forkhead box C1), which encodes a transcription factor and a potential prognostic biomarker specific for a basal-like BC subtype. It should be noted that the majority of TNBC (approximately 80%) are also basal-like BC subtype [2].

Furthermore, we found inverse expression patterns in the JygMC(A) primary tumors as compared to the NMG and ER+ (estrogen receptor positive) subtype. Thus, ESR1 (estrogen receptor 1), which encodes for a hormone-activated transcription factor, was significantly down-regulated in the primary mouse mammary tumors. In contrast, ESR1 was among the top-ranked human genes expressed in ER+ versus ER- BC subtypes in the TCGA dataset. ESR1 up-regulation is associated with ER+ and progesterone receptor (PR)+ BC and is an important prognostic and predictive biomarker [28]. Dataset S4A shows that mouse genes displayed more discordant than concordant expression patterns with genes expressed in ER+. Only 2-5% of up- or down-regulated mouse genes were also increased in the ERBB2- positive subtype. Unfortunately, the lack of large-scale human datasets (other than TCGA) prevented us from establishing the statistical significance of concordantly expressed genes in the mouse and human datasets.

### Genes down-regulated in JygMC(A)-derived lung metastases are also decreased in human BC metastases

To evaluate the relevance of the JygMC(A) mouse model to human metastatic cancer, we compared our data with the three largest human BC studies in the Oncomine datasets that had compared primary human breast tumors and metastases [3, 29] and Bittner expression data (unpublished). Two differentially expressed gene sets (Primary tumor *vs*. NMG up- and down-regulated; FC>=2; *p*-value <0.05) were used as queries in searches against the Oncomine datasets. The TCGA dataset was not included in this analysis because it contained only three metastasis samples. Since the overall number of samples (63-336) and especially the number of metastasis samples (5-9) were low in human datasets, the statistical significance of these findings is presently unknown.

The largest amount of concordance with the human studies was observed for the genes that were significantly down-regulated in lung metastases versus primary tumors in our study (Figure 4E and Dataset S4B). Among the accessed datasets, the Radvanyi had the largest number of concordant genes [29]. Interestingly, some of these genes were also part of the metastatic 79-gene signature observed in solid tumors [30]. The list of overlapping genes included Cyr61, Fhl1, Gsn, Mgp, Myh11, Mylk, Rbpms, Sparcl1, Tcf21 and Lum. The 79-gene signature was defined using a meta-analysis of 18 gene expression datasets in Oncomine comparing distant metastases to primary tumors in various solid tumors.

### microRNA profiling identifies known regulators of EMT

MicroRNAs (miRNAs) are small noncoding RNAs that play important post-transcriptional roles by repressing messenger RNA activity [31]. To characterize miRNA expression in the JygMC(A) mouse model, we conducted targeted profiling of 566 miRNAs using commercially available nCounter^®^ Mouse miRNA Expression Assays (NanoString Technologies). The top differentially expressed miRNAs for each comparison (Carcinoma *vs*. EMT-like, Carcinoma *vs*. NMG, EMT-like *vs*. NMG, lung metastasis *vs*. PT and *in vitro* JygMC(A) 3D-spheres *vs*. the monolayer cells) can be found in Dataset S5A. Interestingly, when comparing adenocarcinoma and EMT-like areas of primary tumors, we found among the top differentially expressed genes members of the miRNA-200 family in epithelial-like cells. The miRNA-200 family is known to regulate Zeb1 and Zeb2, and their expression decrease during EMT [32].

miR-203 was found in adenocarcinoma-like areas (primary tumor and lung metastases) and NMG suggesting that it might positively correlate with differentiation and MET markers and negatively correlate with EMT markers [31]. Furthermore, when comparing adenocarcinoma or EMT-like areas with NMG, we found 53 and 79 down-regulated miRNAs, respectively, and only 10 and 11 up-regulated in malignant tissue samples (adenocarcinoma or EMT-like areas respectively). From those up-regulated miRNAs, 8 of them were common in malignant tissue: miR-9, miR-34c, miR-140, miR-337-5p, miR-1196, miR-1892, miR-2133 and miR-2137. This suggests that a greater proportion of miRNAs were silenced during the malignant transformation.

**Figure 5:**
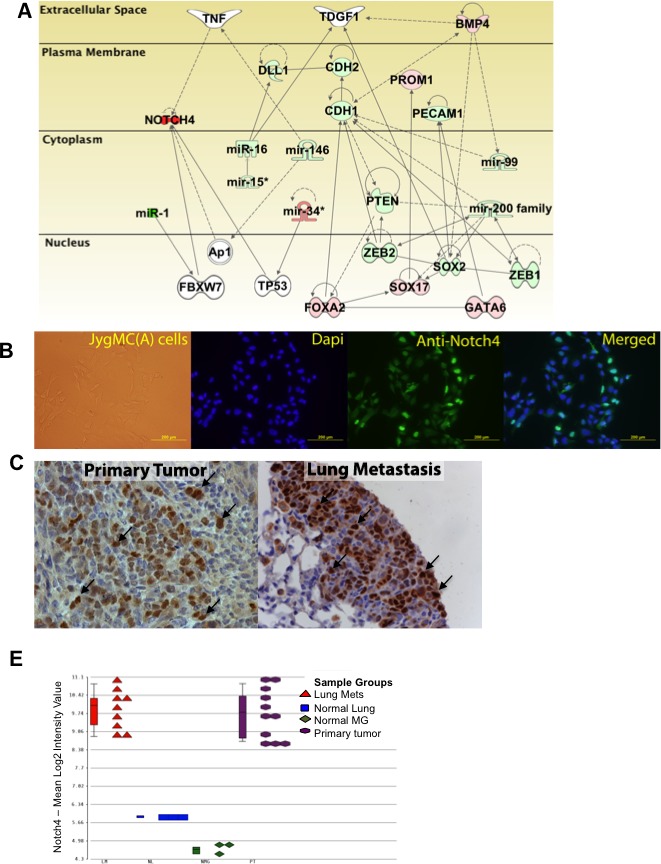
miRNA regulation and Notch4 expression *in vitro* and *in vivo* mRNA and microRNA network in JygMC(A) primary mammary tumor model **B.** Immunofluorescence staining of *Notch4* in adherent JygMC(A) parental cell line **C.** Immunohistochemistry of *Notch4* in primary tumor derived from JygMC(A)-GFP/Luc cells and lung metastasis. Brown staining represents positivity. Arrows indicate nuclear staining. **D.** *Notch4* gene expression throughout microarray samples.

Several members of the miR-15 family, such as miR-15a, miR-16, miR-196, miR-497, as well as miR-205 overexpression, which are known to repress Cripto-1 expression [33, 34], were found in NMG but not in primary JygMC(A) tumors, consistent with potential Cripto-1 involvement in tumorigenesis of mammary tissue. Moreover, we found miRNAs such as miR-1, miR-146, miR-199 and miR-200 family increased in NMG when compared with malignant tissues as well as in primary carcinoma regions when compared with EMT-like regions or lung metastasis (Dataset S5A). A growing body of evidence supports an interrelationship between those miRNAs and the Notch signaling pathway [35]. To further analyze the miRNAs, we used the TargetScan6.2 to compile a list of miRNAs that might be modulating the six validated genes that were found to be differentially expressed in both targeted and global expression analysis (Cdh2, Foxa2, Gata6, Sox17, Prom1 and Bmp4) (Dataset S5B). All of these genes, except Cdh2, are involved in the establishment of lung metastasis through MET. Taken together, these results suggest that a series of microRNAs might permit fine-tuning of the EMT-MET transition in this model. Combining data from mRNA and miRNA analyses results in a proposed signaling network of key genes and regulators, suggesting interaction among Cripto-1/Tdgf-1, Sox2, Bmp4, Cdh1 and miR-15/16, miR-200 family, miR-99; which supports the observations in this detailed study of the JygMC(A) mouse model (Figure 5A).

### Notch signaling has a role in driving tumor growth and metastasis in the JygMC(A) mouse mammary model

We anticipated that the Notch signaling pathway might play an important role in driving tumor growth and metastasis in this system since the NICD was found in the nucleus of the JygMC(A) cells *in vitro* (Figure 5B). In addition, the NOTCH4 protein was also observed in the nuclei of cells in primary tumors and in lung metastases (Figure 5C). Moreover, the observed expression patterns in the microarray showed that Notch4 was overexpressed in the primary tumor tissues and lung metastasis when compared with normal tissue using a microarray platform (Figure 5D).

To verify the potential contribution of Notch signaling during mammary tumor progression, we used the RO4929097 gamma-secretase inhibitor, which is a novel orally-active inhibitor with improved clinical toxicity and currently under investigation in a Phase II clinical trial in treating patients with advanced, metastatic or recurrent TNBC (Trial Registration ID: NCT01151449). In order to assess the effect of this compound on JygMC(A) cells, we performed several cell-based *in vitro* and *in vivo* assays. First, we performed cell viability assays using different concentrations ranging from 2 to 100μM of the RO4929097 inhibitor and equivalent volume of the vehicle controls. Flow cytometry analysis of cell viability using propidium iodide showed no significant cytotoxic effects using 100μM RO4929097 on JygMC(A) cells with 3.48% of cell death and 3.69% of vehicle.

Likewise, the TUNEL assay showed no significant drug-induced apoptotic effects at 100 μM RO4929097 on NMuMG cells (less than a 1%) and 0.86% of apoptotic response for the JygMC(A) cells (a staining representation can be seen on Figure S3A). In order to validate the suppressive effects of the gamma-secretase inhibitor *in vitro*, we verified Notch4 endogenous mRNA levels and whether RO4929097 could reduce Notch downstream effector target genes such as Hes1 by qRT-PCR. As expected, there were no significant changes in total endogenous Notch4 mRNA levels following RO4929097 treatment (Figure S3B). In contrast, we observed a dose-dependent reduction in Hes1 expression suggesting that gamma-secretase is needed for full activation of the pathway (Figure S3C).

Next, we assessed the expression of 9 genes involved in the Notch signaling pathway including receptors (Notch receptors 1-4), downstream target gene effectors (Hey1, Hey2 and Hes5) and one TGF-β family member (Nodal) (Figure S3D). TGF-β signaling collaborates to induce EMT in BC cells and maintain their mesenchymal/CSC states [36]. All genes showed expression mostly *in vivo* and not *in vitro*, except Notch 2, suggesting that the tumor microenvironment favors expression and/or full activation of the Notch signaling pathway and other signaling pathways. The list of genes and oligonucleotide sequences can be found in Table S2.

**Figure 6:**
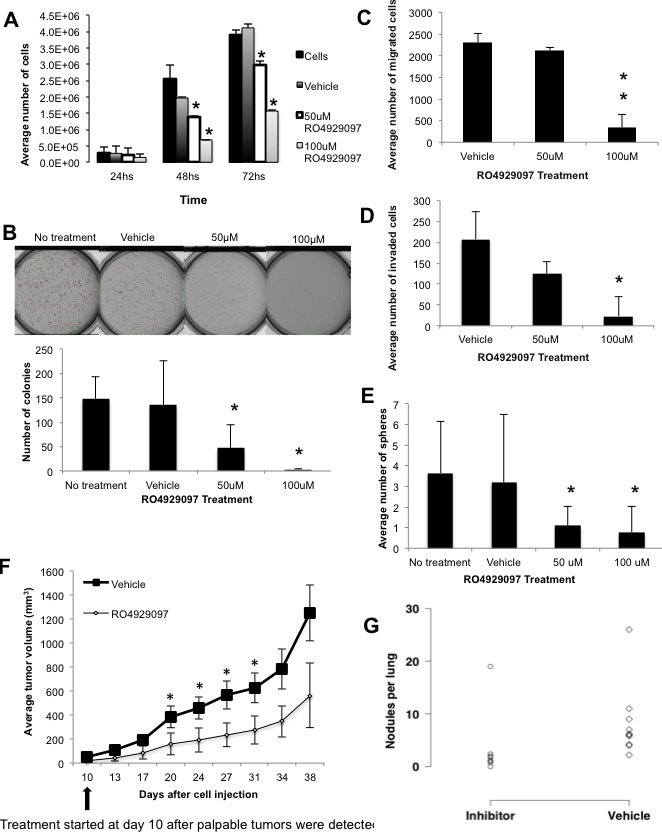
*In vitro* and *In vivo* effects of the gamma-secretase inhibitor RO4929097 in the JygMC(A) cell line and mouse model Proliferation assay. JygMC(A)-GFP/Luc cells were seeded in 12-well dishes in triplicate at 5×10^3^cells/well and cultured for 24, 48 and 72 hrs. Cells were then harvested and counted. Data are representative of two independent experiments in triplicate ±SD, *P < 0.0001, as compared to control vehicle-treated cells. **B.** Colony formation assay in soft agar. A total of 1.5×10^4^cells were cultured for 10 days. Colonies were stained with NitroBlue Tetrazolium and quantified using Gelcount. Data are representative of two independent experiments in triplicate ±SD, *P < 0.005, as compared to control vehicle-treated cells. **C.** Boyden chamber migration and **D.** invasion assays. A total of 4×10^4^cells in serum-free medium were seeded on the top chambers, and 2% or 5% FBS-containing medium was placed in the bottom wells as a chemoattractant. Cells were incubated for 24hrs. Cells that migrated/invaded through the membrane were stained and counted using a light microscope. Data are expressed as the average of cells in ten fields from each membrane (20X). Data are representative of two independent experiments in duplicate ±SD, *P < 0.01 and **P < 0.001, as compared to control vehicle-treated cells. **E.** Sphere formation assay. A total of 10,000 cells were cultured for 10 days on non-adherent plates and treated with different concentrations of RO4929097. Data are representative of two independent experiments in sixplicate ±SD, *P < 0.001, as compared to control vehicle-treated cells. **F.** Nude mice bearing mammary tumors (*n* = 10/group) were dosed orally following the schedule of 60mg/Kg every other week (7days on/7days off) or vehicle for 4 weeks and primary tumor volumes are represented in the graph, ±SD, *P < 0.05, as compared to control vehicle-treated animals. **G.** Number of pulmonary nodules per animal in control vehicle-treated and RO4929097-treated animals (*P < 0.05, one-sided values; Wilcoxon rank-sum test).

Next, we assessed whether the RO4929097 treatment could affect proliferation, soft agar colony formation, cellular migration and/or invasion, and tumorsphere formation of the JygMC(A) cells. In all of these *in vitro* assays, RO4929097 significantly inhibited the biological responses. For example, after 48hrs of the RO4929097 treatment, we observed approximately 30% and 62.5% inhibition in proliferation using 50μM and 100μM of RO4929097, respectively, as compared with vehicle-treated control cells (Figure 6A). A reduction of 60% in the number of soft-agar colonies was found when assessing anchorage-independent growth using 50μM of RO4929097 (Figure 6B). A significant decrease (around 70%) in migration and invasion was found with 100μM RO4929097 treatment after 24hrs (Figure 6C-6D). Treatment with 50μM RO4929097 also significantly impaired tumorsphere formation (Figure 6E). Moreover, RO4929097 significantly inhibited primary tumor growth during treatment; however, the inhibitory response of the drug was time limited as its potency was reduced at later time points as tumors increased in size (Figure 6F). A reduced number of metastatic lung nodules were observed in animals treated with RO4929097 as compared with vehicle-treated animals (Figure 6G).

### Cripto-1 promoter is active during primary tumor growth but not in metastasis

**Figure 7:**
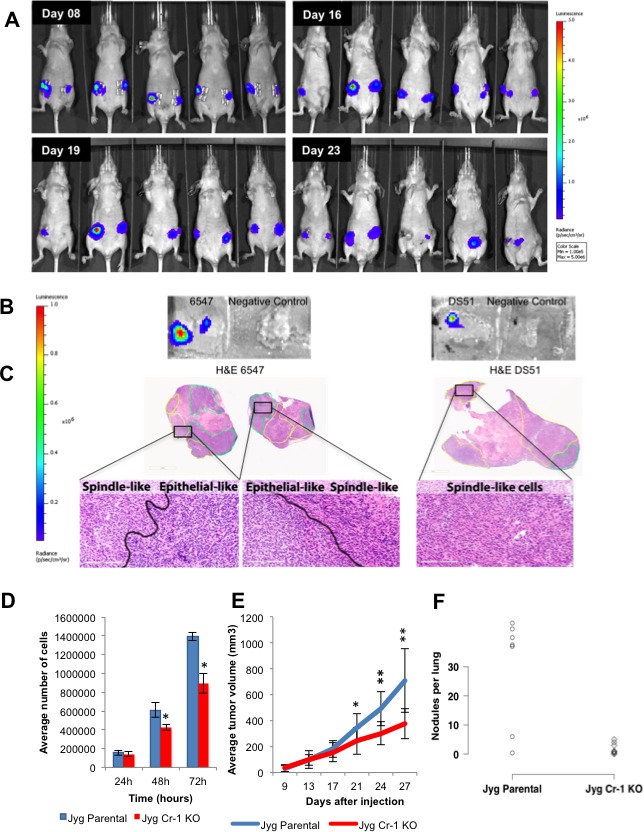
Mouse Cripto-1 promoter and Cripto-1 knockout in JygMC(A) cell line Representation of bioluminescent imaging on animals injected bilaterally into the fourth mammary gland with JygMC(A) cells containing the mouse *Cripto-1* promoter. Animals were imaged at day 8, 16, 19 and 23-post cell injection. **B.** *In situ* detection of *Cripto-1* promoter activity in primary tumor tissue sections. **C.** H&E of the primary tumor tissue sections depicted in **B.** showing carcinoma areas (green) and EMT-like areas (yellow) and high magnification (20X). Scale bars: 3mm and 200μm. **D.** Proliferation assay. JygMC(A) cells were seeded in 12-well dishes in triplicate at 5_x_10^4^cells/well and cultured for 24, 48 and 72 hrs. Cells were then harvested and counted. Data are representative of two independent experiments in triplicate ±SD, *P < 0.0002, as compared to control cells. **E.** Average of primary tumor volumes are represented in the graph on JygCr-1KO and JygMC(A) parental cells (*n* = 7 animals/group ±SD, *P < 0.01 and **P < 0.0001, as compared to control animals). **F.** Number of pulmonary nodules per animal in JygCr-1KO and JygMC(A) parental animals (*P < 0.05, one-sided values; Wilcoxon rank-sum test).

Since CRIPTO-1 physically interacts with all four Notch receptors and Notch4 and Cripto-1 has been shown to be relevant in embryogenesis, maintenance of a human BC stem cell population and tumorigenesis [6, 13, 14], we decided to investigate the role of Cripto-1 in the JygMC(A) mouse model. We generated a reporter system using the luciferase gene driven by the promoter for the mouse Cripto-1 gene. The mouse Cripto-1 promoter sequence can be found in Table S3. The reporter can detect real-time Cripto-1 expression *in vitro* and *in vivo* during tumor growth and lung metastasis. To validate the activity of the mouse Cripto-1 luciferase reporter *in vitro*, we performed a dual-luciferase assay using mouse F9 embryonal carcinoma cells that express high levels of endogenous Cripto-1 [37]. To induce the Cripto-1 promoter activity, we used two TGF-β-related family members: TGF-β1 and NODAL which were previously reported to activate the human Cripto-1 promoter [37] (Figure S4A and S4B). After construct validation, stably transfected JygMC(A) cells containing the mouse Cripto-1 promoter reporter construct or the negative-control were injected bilaterally into the fourth mammary fat pads (*n* = 5 animals/group). Tumor growth was assessed twice a week for the Cripto-1 promoter activity and weekly for negative-control animals. Using this unique detection system, we determined that the Cripto-1 promoter was expressed in 100% (10/10) of the injection sites of primary JygMC(A) mammary tumors (Figure 7A), whereas the same promoter construct was silenced in pulmonary metastases. This confirmed that this embryonic stem cell gene is only active during primary tumor growth and during the initiation of metastasis.

We then wanted to validate NanoString-based evidence that the Cripto-1 gene is expressed in EMT-like areas of primary tumors. To ascertain this, we performed an *in situ* luciferase expression assay on primary tumors derived from JygMC(A)-Cripto-1 promoter fluorescently-tagged cells. Living Image *In Vivo* Software was used to identify localized sites in the primary tumor sections showing luminescence (Figure 7B). Even though the primary tumors possessed a mixed cell population (adenocarcinoma and EMT-like areas) based on H&E staining, Cripto-1 promoter activity was observed mostly in the EMT-like areas (Figure 7C). These results are consistent with other studies suggesting that Cripto-1 plays a role in the induction of an EMT program and might support the reprogramming of differentiated tumor cells into CSC.

### *Cripto-1* knockout inhibited proliferation *in vitro* and tumor volume *in vivo*

In order to investigate the role of Cripto-1 in this mouse model, we generated JygMC(A) mutant cells using the double nicking RNA-guided Cas9 nucleases from the microbial CRISPR (clustered regularly interspaced short palindromic repeat)-Cas system. After isolation of clonal cells, a homozygous mutant clone (JygCr-1KO) was confirmed by sequencing (Figure S5). A 35% reduction in cell growth was observed after 72 hrs for JygCr-1KO cells as compared with JygMC(A) parental cells (Figure 7D). More importantly, animals that received JygCr-1KO cells into both fourth mammary fat pads showed approximately 45% reduced primary tumor volume and reduced number of metastatic lung nodules when compared with JygMC(A) parental cells (Figure 7E and 7F, respectively), suggesting that Cripto-1 plays a significant role during tumor growth and metastasis in the JygMC(A) mouse mammary model.

## DISCUSSION

Pre-clinical animal models that can recapitulate the metastatic process and resemble specific molecular subtypes of human BC as well as facilitate identification and validation of candidate drug target genes are greatly desired since gene expression patterns in the mouse models shows extraordinarily significant correlations with those of the human conditions [38]. Here we show a comprehensive molecular analysis of a spontaneous mouse model of metastatic mammary carcinoma in a feral outbred strain of mice that developed spontaneous mammary carcinoma. It is similar to the lymphatic human BC metastatic process that phenotypically and molecularly resembles the human TNBC subtype, which is associated with the poorest prognosis due to its high resistance to chemo- and radiotherapy [2].

Using the Oncomine integrated data-mining platform, we found similarities between the JygMC(A) mouse model and the human TNBC molecular subtype at the gene expression level. Moreover, when comparing the metastatic gene expression pattern between the JygMC(A) metastatic process and human BC studies, we found that the greatest concordance was observed for genes that were significantly down-regulated in lung metastases. A similar observation was made by Daves and colleagues, who proposed that overcoming metastatic suppression might be a critical feature in metastatic tumors [30].

Evidence for phenotypic plasticity in aggressive TNBC has been reported [8]. It is well established that EMT is a process characterized by loss of cell adhesion, increased cell motility and acquisition of a mesenchymal phenotype, whereas MET represents a reverse process that involves the transition from motile, spindle-shaped mesenchymal cells to planar arrays of polarized epithelial cells [4]. Here we demonstrated spontaneous epithelial-mesenchymal plasticity in JygMC(A) primary mammary tumors and lung metastasis. Histological phenotyping showed two types of cell morphology suggesting cell plasticity: (i) an undifferentiated metaplastic mammary adenocarcinoma cell type that might transition into a more (ii) spindle-like mesenchymal cell type with EMT characteristics, which could then facilitate migration and invasion into adjacent tissues. In contrast, lung metastasis lesions exhibited an epithelial phenotype only, which suggests that the reverse biological process, MET, might allow metastasizing mesenchymal cells to regain epithelial properties, facilitating the colonization of a new environment or niche.

When comparing the adenocarcinoma areas in the primary tumor and lung metastasis, we observed distinct gene expression profiles. Even though they exhibited a similar morphology, we found differences at the molecular level, which suggests that the primary tumor adenocarcinoma already exhibits a metaplastic signature partially similar to the cells undergoing EMT. A similar observation was made in human BC during the progression of ductal carcinoma *in situ* where molecular changes in human cells occurred before morphological alterations [39]. Cell adhesion and migration were among the most important biological processes affected by these molecular changes [39].

Recent studies have also shown a molecular signature in TNBC that is highly correlated with the activation of the XBP1 branch of the unfolded protein response pathway and hypoxia sustaining CSC maintenance [9]. In our TNBC model, we found up-regulation of Pou5F1 (Oct-3/4), Kit, Cd24 and Itga6 (Cd49f) in epithelial-like adenocarcinoma areas of primary tumors, while Klf4, Sox2, Cripto-1 were expressed in the mesenchymal/EMT-like regions. The enrichment of Cripto-1 expression in basal-like human breast cancer has been reported by our group [40]. Cripto-1 has been shown to be involved in tumor epithelial cell plasticity and may be an important EMT regulator together with downstream genes such as Snail, Slug, Twist, and Six1 [11]. Since Cripto-1 has been demonstrated to promote EMT *in vitro* in mouse mammary epithelial cells and *in vivo* in MMTV-Cripto-1 and WAP-Cripto-1 transgenic mouse mammary tumors [6, 41], it is reasonable to hypothesize that Cripto-1 might support self-renewal, invasiveness and metastatic abilities of BC stem-like cells through the induction of an EMT program. This seems possible since Cripto-1 is expressed in a subpopulation of mouse mammary epithelial cells that has stem cell-like or progenitor properties [42]. We found no or very low Cripto-1 levels in JygMC(A) cells in culture, suggesting that the microenvironment might trigger Cripto-1 expression *in vivo*. Interestingly, Nodal a TGF-β family member and ligand that binds to Cripto-1 activate a Smad2/3 signaling pathway also exhibited a similar pattern of expression as Cripto-1. In addition, knock out of the Cripto-1 gene in JygMC(A) cells via the CRISPR-Cas9 editing system resulted in significant tumor growth inhibition and a pronounced reduction in pulmonary metastasis, suggesting that Cripto-1 may contribute to mammary oncogenesis in this setting.

Cripto-1 promoter activity was detected during primary tumor growth in the EMT-like regions but not in the lung metastases since no EMT-like areas were observed in the lung metastasis. One possible explanation is that the Cripto-1-associated pathway might have become Cripto-1-independent during metastasis. Alternatively, this might be due to diminished expression of Cripto-1 receptors or ligands, such as Grp78 or Nodal, respectively, in the lung metastases. Still, the mechanism by which Cripto-1 is signaling in the primary mammary tumors is unknown and could involve either SMAD-dependent or SMAD-independent pathway [6, 43].

Bmp4, Foxa2, Sox17, Prom1 and Gata6 were expressed genes that were common detected in both the targeted (NanoString) and global (Affymetrix) gene expression platforms and showed overexpression in the lung metastases. Bmp4 has been shown to produce a dramatic reduction in Cripto-1 promoter activity in NTERA-2 and LS174-T cells [37]. Also, Teo and collaborators showed that Bmp4 represses pluripotent gene expression, such as Oct-4 and Sox2, and accelerates differentiation [44]. For instance, BMP4 enhances ACTIVIN A-induced definitive endoderm differentiation leading to an increased expression of Cxcr4, Sox17 and Foxa2, suggesting that Bmp4 might regulate those genes during lung metastasis colonization [44] and block Cripto-1 expression [37]. Finally, Bmp-4 has been demonstrated to be necessary in the maintenance an epithelial phenotype in BC lung metastases [22].

Intrinsic regulators of gene expression, such as the miR-200 family, were found in adenocarcinomas but not in the EMT-like areas. This confirms previous findings that the EMT process is dependent on expression of the miRNA-200 family, which is decreased during EMT [32]. In addition, miR-203 was up-regulated in adenocarcinoma-like areas (primary mammary tumor and lung metastases) and NMG suggesting that it might positively correlate with markers of differentiation and MET. These results are in agreement with studies showing that miR-203 expression is inversely correlated with BC stemness and EMT markers and its epigenetic silencing was required for EMT [31, 45]. Moreover, we found that down-regulation of the miRNA15a-16 family and miR-205 was observed in primary tumors when compared to NMG, which agrees with previous findings that these microRNAs can repress Cripto-1 expression and can inhibit non-small cell lung cancer progression and restore chemotherapeutic drug sensitivity [33, 34].

Finally, since Notch4 has been reported to play a role in TNBC [46] and is expressed in a population of tumor-initiating stem cells [47], we demonstrated that Notch inhibition might be an effective strategy for the treatment of BC, at least in an animal model that resembles human TNBC. Treatment with the gamma-secretase inhibitor RO4929097 decreased primary tumor growth and significantly reduced the number of metastatic lung nodules. Similar observations using RO4929097 and other gamma-secretase inhibitors have been made using human TNBC lines [47-49]. However, a more specific target might be necessary since gamma-secretase inhibitors do not exclusively target the Notch signaling pathway [50]. Moreover, no significant changes were observed in primary mammary tumor histology for gamma-secretase inhibitor-treated animals when compared with non-treated animals.

In summary, our findings strongly suggest that Cripto-1 is a potential driver of mammary tumorigenesis, and offers evidence of spontaneous epithelial-mesenchymal plasticity in a novel metastatic mammary tumor model that phenotypically and molecularly resembles the human TNBC subtype. As Notch signaling interacts with multiple other pathways that include candidate therapeutic targets, understanding these interactions will greatly benefit rational design of combinatorial therapeutic regimens. We propose a novel potential therapeutic approach that involves targeting proteins such as Cripto-1, involved in EMT and CSC maintenance in BC. This may lead to a more efficient therapeutic strategy for the treatment of TNBC in particular, since Notch receptors, an EMT phenotype and Cripto-1 expression have all been found in TNBC. Moreover, this novel TNBC mouse model can be used to screen for novel compounds in the context of signaling pathways having implications in the initiation and progression of TNBC.

## MATERIALS AND METHODS

More detailed methods can be found in Supporting Information.

### Cell line, culture conditions and reagents

Cells were confirmed to be free of mycoplasma contamination using PCR (Mycoplasma Kit, Promokine). Mouse mammary carcinoma cell line JygMC(A) was kindly provided by Dr. Shogo Ehata (University of Tokyo, Tokyo, Japan) and were maintained in Dulbecco's Modified Eagle Medium (DMEM 1X; Gibco/Life Technologies, Grand Island, NY), supplemented with 10% Fetal Bovine Serum (FBS) and 100 units/ml penicillin, and 100 μg/ml streptomycin at 37°C in 5% CO2. F9 embryonal carcinoma cells (ATCC^®^ CRL1720™) and the non-transformed mouse mammary epithelial cell line NMuMG (ATCC^®^ CRL1636™) were purchased from ATCC (Manassas, VA) The F9 cells were maintained on gelatin coated cell culture plates. The RO4929097-001 gamma-secretase inhibitor and vehicle were generously provided by Hoffmann-La Roche (Nutley, NJ) (DCTD-CTEP/NIH agreement) already in suspension.

### Establishment of stable JygMC(A)-GFP/Luc mammary carcinoma cell line

JygMC(A) cells were transduced with a lentivirus vector containing a fusion construct of the firefly luciferase and eGFP reporters (Leidos/FNLCR, Frederick, MD). The transduced cells were enriched in medium containing 200 μg/mL Geneticin (Invitrogen, Grand Island, NY) for 10 days and sorted by FACs to select for a pure population using the BD FACSVantage™ (BD Bioscience, San Jose, CA).

### Mouse strain and animal care

Animals used in this study were female Balb/C nude mice aged 6-9 weeks (National Cancer Institute). Animal procedures were conducted under conditions approved by the Frederick National Laboratory for Cancer Research (FNLCR), an Association for Assessment and Accreditation of Laboratory Animal Care International (AAALAC) accredited institution that follows the Public Health Service Policy for the Care and Use of Laboratory Animals outlined in the “Guide for Care and Use of Laboratory Animals”; FNLCR ACUC 11-067 approval on 03/16/2012.

### Orthotopic mammary fat pad injection and mammary tumor formation

Mouse mammary carcinoma cells were grown to approximately 75% confluence. Balb/C athymic nude mice were anesthetized with isofluorane/O2 and injected bilaterally into the fourth mammary fat pad with 20μl of 5×104 cells/gland or as described otherwise. Cells were suspended in 25% Growth Factor Reduced Matrigel (BD Bioscience, Bedford, MA), containing 10% Trypan Blue Stain (Lonza, Walkersville, MD) and 65% PBS. Tumor growth from both sides were measured twice a week and monitored weekly by *in vivo* bioluminescent imaging. Tumor volume was calculated using the formula: ½ (LxWxD), where l=length, w = width and d= depth. At 20-30 days after cell injection, mammary primary tumors were removed as a parallel of the human clinical setting. Ten days later, animals were imaged and metastases in lungs were observed over a period of 4-6 weeks. Statistical analysis of differences in tumor volume was performed using Student t-test (GraphPad Software, Inc. 2014 – online version).

### Bioluminescent imaging

Bioluminescence was performed using a Xenogen IVIS-100 imager (PerkinElmer, Waltham, MA). Images were quantified as total photon counts or photons using Living Image^®^ software (PerkinElmer, Waltham, MA)

### *In situ* detection

Frozen OCT-embedded tissue blocks were cut using a cryostat into 10-μm sections as previously reported by Roberts and collaborators [51]. Slide-mounted tissue sections received a few drops of luciferase assay substrate (Luciferase Assay System, Promega Corporation, Madison, WI) and images were performed within 5 minutes.

### In vivo

Animals were injected with the substrate D-Luciferin Potassium salt (PerkinElmer, Waltham, MA) in Phosphate Buffered Saline (PBS; Invitrogen, Grand Island, NY) by intraperitoneal injection at 150 mg/Kg. The animals were anesthetized with isofluorane/O2 and placed in the IVIS™ Imaging System. Images were taken 15min after luciferin injection from the ventral view.

### Sphere formation assay

Assay was performed as previously reported [40]. For the second and third-generation spheres, previous generation were collected and centrifuged at 350xg for 5 minutes. The pellet was resuspended in 1mL of pre-warmed Trypisin-EDTA (Catalog # 07901, Stem Cell Technologies, Vancouver, Canada) and spheres were disrupted by pipetting to get single cell suspension. The same density of cells was plated and cultured for 10 days. Third-generation spheres were used in all experiments.

### TUNEL assay

Cells were grown in Lab-Tek chamber slides (Nalge Nunc, Rochester, NY). 1×104 NMuMG or JygMC(A) cells were seeded and RO4929097 treatment was added on the following day. Cells were incubated for 48hrs following staining using the *In Situ* Cell Death Detection Kit, Fluorescein, (Roche, Mannheim, Germany) in accordance to manufacturer instructions. Cells were counted from ten different fields.

### Laser capture microdissection, targeted microRNA and mRNA gene expression profiling using NanoString assays

Cells were laser captured using the MMI CellCut Plus (Molecular Machines & Industries, Glattbrugg Switzerland) as previously described [52] and lysed using the lysis buffer RLT from Qiagen Micro RNAeasy Kit (Qiagen, Gaithersburg, MD). For microRNA analysis, total RNA lysates from cells were purified using the miRNeasy Micro Kit (Qiagen, Gaithersburg, MD) in accordance with manufacturer instructions. The mRNA and miRNA data have both been deposited in NCBI's Gene Expression Omnibus (GEO) and are accessible through GEO Series accession number GSE63627 and GSE63633, respectively.

### Microarray data processing

Microarray analysis was done using Partek Genomic Suite version 6.6 (Partek, Inc.). Microarray CEL files were pre-processed using RMA (Robust Multi-Array Average) background correction and quartile normalization to correct for array biases. Probe intensities were log-transformed and summarized using Median Polish. One sample (6518LM) was excluded from further analysis because it failed multiple quality metrics. After Batch Remove, a standard One-way ANOVA analysis was then performed with tissue type as factor to identify differentially expressed genes (FDR corrected *p*-value <0.05 and FC cutoff ≤2 unless indicated otherwise). Microarray data in this publication have been deposited in NCBI's Gene Expression Omnibus (GEO) and are accessible through GEO Series accession number GSE63951. Venn diagrams were generated by Partek Genome Suite 6.6 (PGS, Version 6.6, Partek, Inc.).

### Pathway enrichment analysis

To identify biological characteristics of the differentially expressed genes, we used the DAVID annotation tool to identify over-represented KEGG pathways for a given set of differentially expressed genes from the microarray analysis. The KEGG pathways were ranked and filtered based on the Benjamini-Hochberg *p*-value <0.05. The topmost enriched pathways are represented for primary tumors versus NMG and lung metastasis versus primary tumors with the number of genes being plotted for that particular pathway.

### RNA extraction protocol

For qRT-PCR and global gene expression analysis, all RNA tissue samples were snap frozen in liquid nitrogen. Total RNA from tissue samples was extracted using TRIzol^®^ reagent according to the manufacturer's recommendations (Invitrogen, Carlsbad, CA). Total RNA from cells or spheres was isolated and purified using the RNeasy Mini Kit and subjected to DNAse treatment (Qiagen, Gaithersburg, MD) in accordance to manufacturer instructions. Following extraction, 1μg of total RNA was reverse transcribed using the RETROscript^®^ kit (Ambion, Carlsbad, CA) in accordance with manufacturer's instructions.

### Oncomine analysis (meta-analysis)

Concordance analysis between mouse mammary carcinoma and human BC expression data-sets was performed on selected BC gene (mRNA) expression datasets in Oncomine (Oncomine v4.5 Research Edition, www.oncomine.org [23].

### Immnunofluorescence

Primary antibodies used include: NOTCH4 receptor (1:200 dilution, Millipore), anti-CK18 (1:200 dilution, Abcam), anti-K-5 and anti-K14 (1:200 dilution, Covance), anti-CDH1 and anti-VIM (1:50, Santa Cruz Biotechnology).

### Immunohistochemistry

Primary antibodies used were Estrogen Receptor-alpha M20, Santa Cruz Biotechnology 1:200; PR, polyclonal rabbit A0098, Dako 1:200; CDH1, mouse monoclonal M3612, Dako 1:500; BMP-4, rabbit polyclonal, ab39973 Abcam 1:100, HER2, rabbit monoclonal, D8F12, Cell Signaling 1:200; NOTCH4 07-189, Millipore, 1:1000, HEY-1 ab 5714 Millipore, 1:1000.

### Construction of the mouse Cripto-1 promoter driving expression of the firefly luciferase reporter gene

A multisite gateway entry clone containing the 2.6 Kb mouse tdgf-1/cripto-1 promoter sequence (2.2kb from ATG start site) was cloned from a BAC DNA into a vector composed of a fusion of the firefly luciferase and eGFP reporters.

### Generation of JygMC(A) mutant cells using CRISPR-Cas9n

Generation of JygMC(A) mutant cells using CRISPR-Cas9n was performed as reported by Ran and collaborators [53]. A homozygous mutant clone (Jyg Cr-1KO) containing a 137bp deletion was confirmed by sequencing and used for further study.

### Statistical analysis

Results are expressed as standard deviation if not otherwise indicated. Values of *p* ≤ 0.05 were considered statistically significant, as determined by the ANOVA test, the two-tailed unpaired Student *t* test, or the Wilcoxon rank-sum rank test where appropriate.

## SUPPLEMENTARY MATERIAL, FIGURES AND TABLES












