# Supplementary material for: Cripto-1 as a novel therapeutic target for triple negative breast cancer
Source: Oncotarget. 2014 May 19;6(14):11910–29. doi: 10.18632/oncotarget.4182 (PMC4494913; doi:10.18632/oncotarget.4182)
Supplement: Supplementary file 1 [file oncotarget-06-11910-s001.pdf]

Cripto-1 as a novel therapeutic target for triple negative breast cancer

Supplementary Material

Supplementary Methods

Quantitative Real Time PCR (qRT-PCR)

qRT-PCR reactions for 21 mouse genes were performed in 2-7 samples using the Mx3005P (Stratagene, La Jolla, CA) and carried out using Brilliant Untra-Fast SYBR® Green QPCR MasterMix (Agilent Technologies, Santa Clara, CA) in a total volume of 20μl using the Fast program. To evaluate the amplification of nonspecific products and primer-dimer formation, dissociation curves were analyzed. The reactions were performed in duplicate. Two internal control genes, *Hprt1* [GenBank:NM_000194] and *Gapdh* [GenBank:AJ005371] were considered in gene expression normalization. Relative gene expression between sample groups was calculated using the Pfaffl model [1], employing the efficiency-corrected equation. The list of genes and oligonucleotide sequences can be found in Supplementary Table S7.

Laser Capture Microdissection (LCM), targeted microRNA and mRNA gene expression profiling using NanoString assays

Total RNA lysates (36 samples) and purified RNA (24 samples) were prepared for mRNA and microRNA NanoStrings analysis, respectively. Based on published literature, 103 mRNA genes (Supplementary Table S2) and controls classified as ESC, EMT and MET markers were selected for nCounter® Custom Gene Expression Assay (NanoStrings Technologies). For the miRNA assay, the

commercially available nCounter® Mouse miRNA Expression Assay (NanoStrings Technologies) containing 566 microRNAs and controls were used. Both assays were performed according to the manufacturer's instructions (NanoStrings Technologies, Seattle, WA).

For mRNA data, normalization was performed using the software nSolver version 1.1 (NanoStrings Technologies). The normalized counts were imported into the MultiExperiment Viewer Version 10.2 (MeV_4_8, http://www.tm4.org) software [2]. Probe intensities were median-centered and log-transformed. Significant transcripts were selected using a *t-test* or the ANOVA statistical test with $p < 0.01$ or 0.05. For clustering samples based on gene expression profile, we applied an unsupervised hierarchical clustering using Pearson's correlation and average linkage.

Genome-wide Affymetrix microarray hybridization

For genome-wide gene expression profiling, all cDNA samples were processed using the GeneChip HT 3' IVT Express Kit (Affymetrix, Santa Clara, California) according to the manufacturer's protocol. Samples were then hybridized to the Affymetrix GeneChip Mouse Genome 430 2.0 Array (platform accession number GPL1261) in the Affymetrix GeneChip Hybridization Oven 640 for 16hrs according to the manufacturer's recommendations (Affymetrix). Arrays were scanned using Affymetrix GeneChip Scanner 3000 7G and processed using GeneChip® Command Console Software (AGCC).

Oncomine Analysis (meta-analysis)

Expression data were median-centered and log2-transformed across samples. Genes were ranked by p-value for every analysis with lower ranking genes having more significant p-values. FC estimates represent the difference between mean values of the classes compared of Oncomine differential analyses. Differential expression was identified by a permutation test with shrinkage to reduce the noise in the data [3]. For each mouse gene set obtained in our study, we extracted gene ranks, p-values, and median FC estimates with no threshold for gene rank or p-value from human breast cancer studies using criteria outlined in the next paragraph. A limited number of mouse genes in our datasets that did not have a matching human gene symbol (HUGO) used in the Oncomine database were excluded from this meta-analysis, so only orthologous genes that have a common mouse and human identifier were used.

In the molecular subtype analysis, we searched Oncomine using genes differentially expressed in JygMC(A) primary mammary tumors and normal mammary glands as a query (FDR adjusted p-value <0.05 and FC cutoff ≤3). To minimize the number of spurious hits in the BC subtype analysis, the following eligibility criteria were applied to Oncomine datasets: (1) generated from human gene expression (mRNA) studies that compared molecular subtypes of invasive breast cancer; (2) had at least 151 samples or more, and (3) contained at least six samples in each subtype group. Using these criteria, five published human breast

cancer studies [4-8] and TCGA were identified. Curtis and collaborators study was excluded because their subtypes were too granular.

In the metastasis analysis, genes differentially expressed in JygMC(A) primary mammary tumors and metastases were used as a query (FDR adjusted p-value <0.05 and FC cutoff ≤2). To select human datasets, the following criteria was -should be were used: (1) generated from human breast cancer studies that included metastasis and primary samples; (2) had at least 50 samples total, and (3) contained at least five samples in each group, in which three datasets were identified [9, 10] and Bittner expression data (unpublished).The TCGA dataset was not included in the analysis because it contained only three metastasis samples.

Immnunofluorescence

Immunostaining was performed on cultured cells grown in Lab-Tek chamber slides (Nalge Nunc, Rochester, NY). Cells were fixed in 4% paraformaldehyde, permeabilized (0.5% Triton X-100 in PBS) and incubated in blocking buffer (10% normal serum from the same species as the secondary antibody in 0.1% Bovine serum albumin (BSA) in PBS) for 60 min at room temperature. The cells were than incubated with the primary antibodies overnight at 4°C. Fluorescently-conjugated secondary antibodies were used to detect the binding of primary antibodies (1:1000 dilution, Invitrogen, Grand Island, NY) and nuclei were stained

with Fluoro-Gel II, with DAPI (Electron Microscopy Sciences, Hatfield, PA). The immunofluorescent samples were visualized using an Olympus BX40 fluorescence microscope and the images were acquired using the camera Olympus DP70 and the software DP Controller 2.1.1.183 (Olympus Corporation, Japan).

Immunohistochemistry (IHC) staining and scoring

Formalin-fixed, paraffin-embedded mouse tissues were sectioned followed by deparaffinization in xylene, ethanol rehydration, and antigen retrieval using 1x Target Retrieval Solution (DAKO, Carpinteria, CA). Four blocking steps were applied to all of the sectioned tissue samples: endogenous peroxidase using 3% hydrogen peroxide (Fisher Scientific, Fair Lawn, NJ) diluted in ethanol; avidin and biotin blocking using the Avidin/Biotin blocking kit (Vector Laboratories, Burlingame, CA) to reduce background, blocking buffer using 10% normal serum from the same species as the secondary antibody and 10% BSA in PBS. Staining was performed using the Vectastain Elite ABC System (Vector Laboratories, Burlingame, CA) according to manufacturer's instructions. Color detection was produced with diamino benzadine peroxidase substrate kit (Vector Laboratories) and sections were counterstained with hematoxylin (Vector Laboratories). Negative control reactions were conducted using the same procedure without the use of the primary antibodies. CDH1 and BMP-4 were detected with Dako EnVision^{TM}+. The presence of a clearly visible dark brown precipitation was considered as immunopositive.

Tissue Microarray platform (TMA) assay and statistical analysis

Core biopsies were extracted from previously defined areas of the primary mouse tissue using a Tissue Microarrayer MTA-1 (Beecher Instruments®, Silver Springs, USA). Tissue cores with a dimension of 0.6 mm from each specimen were punched and arrayed on a recipient paraffin block sampled in duplicates or quadruplicates depending on the availability of the tissue. Slides were prepared from the recipient block using traditional microtomy. The custom tissue microarray was built by Leidos/NCI-Frederick, MD and comprised of 1-2 normal specimens (normal mammary gland, normal lymph nodes, normal lung parenchyma), 6 cases of primary tumors (6 adenocarcinoma and 4 EMT regions), 3 specimens of lymph node metastases (3 adenocarcinoma and 3 EMT regions) and 6 corresponding lungs metastases. The percentage of positive nuclei was calculated in two-four sections. Based on staining intensity, the tissue cores were classified as negative/weak (0,1) or positive/strong (2,3) for BMP-4 and CDH1.

***In vitro* and *In vivo* experiments using the gamma-secretase inhibitor RO4929097**

In vitro: JygMC(A)-GFP/Luc cells were treated with different concentrations of RO4929097 ranging from 2 to 100μM. **Cell viability** was accessed using propidium iodide (Invitrogen, Grand Island, NY) according to the manufacturer's instructions by FACs analysis. Also, quantification of apoptosis was verified using *In Situ* Cell Death Detection Kit (ROCHE, Mannheim, Germany) in accordance to

manufacturer instructions. **Cell proliferation assay:** JygMC(A)-GFP/Luc cells were seeded into a 12-well dish in triplicate at $5x10^4$ cells/well and cultured for 24, 48 and 72hrs. Cells were then harvested and counted using hemocytometer. **Colony Formation assay**: a total of 1.5x104 cells were cultured for 10 days in soft agar to access anchorage-independent growth. Colonies were stained with NitroBlue Tetrazolium and quantified using the Gel Count TM (Oxford OPTRONIX version 1.03). **Boyden chamber migration and invasion assays:** a total of 4x104 cells in serum-free medium were seeded on the top chambers of BD Control Inserts or BD Matrigel coated invasion chambers respectively (BD Biosciences, Bedford, MA). Five percent FBS-containing medium was placed in the bottom wells as a chemoattractant. Cells were incubated for 24hrs. Cells that migrated/invaded through the membrane were stained with Protocol HEMA3® stain set (Fisher Scientific Company, Kalamazoo, MI) and counted using a light microscope. Data were represented as the average of cells in ten fields from each membrane (20X). ***In vivo:*** RO4929097 treatments started at day 10-post cell injection in the mammary fat pad and after palpable tumors were detected. Vehicle or RO4929097 was administered orally to 10 mice per group at 60 mg/Kg for 7 days on and 7 days off for 4 weeks (as previously reported by Luistro and collaborators) [11]. The Wilcoxon rank-sum test was used to determine the significance in differences in numbers of pulmonary nodules between the two groups. A one-sided p-value was presented.

Construction of the mouse *Cripto-1* promoter driving expression of the firefly luciferase reporter gene and *in vitro* validation of activity

Mouse *Cripto-1/Tdgf-1* promoter - ffLuc2-eGFP reporter or control no promoter (-ffLuc2-eGFP reporter) were designed by Leidos/FNLCR. JygMC(A) parental mouse mammary carcinoma cells were transfected using the X-tremeGene HD DNA transfection reagent (Roche, Mannheim, Germany). The transfected cells were selected in medium containing 500 μg/mL Geneticin (Invitrogen, Grand Island, NY) for at least 10 days. For *in vitro* validation of the construct, F9 embryonal carcinoma cells ($1x10^5$ cells/well in 12-well/plates) were transfected with 1 μg of *mCripto-1/Tdgf1* promoter reporter construct or the negative control construct using X-tremeGene HD DNA transfection reagent. The pEF1α-Renilla control vector was co-transfected into all cells to normalize for transfection efficiency. Approximately 6hrs after transfection, serum-free medium containing TGFβ1 (10ng/mL) or Nodal (100ng/mL) recombinant proteins (R&D Systems, Minneapolis, MN) were added to the wells for 48hrs. The cells were then lysed and luciferase activity was measured using the Dual-Luciferase Reporter Assay Kit (Promega) according to the manufacturer's instruction.

Generation of JygMC(A) mutant cells using CRISPR-Cas9n and *In vitro* and *In vivo* experiments

The CRISPR Design Tool (http://tools.genome-engineering.org/) was used to identify suitable target sites in a genomic sequence of interest (mouse *Cripto-1* gene). We used the D10A mutant of Cas9 nuclease (Cas9n) that can be targeted

on specific DNA sequences by combining with single-guide RNA (sgRNA) that has complementary sequences of the strand to be nicked. Simultaneous introduction of a pair of closely spaced sgRNAs for both the DNA strands can generate single-stranded nicks on both strands. Closely spaced double nicking induces the double-strand repair pathway of cells and repair via nonhomologous end joining results in the insertion or deletion of bases in the DNA [12]. Each sgRNA expression construct was prepared by cloning sgRNA into expression plasmid px462. After co-transfecting both sgRNA expression plasmids clonal cells were isolated and sequenced. A homozygous mutant clone (JygCr-1KO) containing a 137bp deletion was confirmed by sequencing and used for further study.

In Vitro**:** For cell proliferation assay**,** JygMC(A) parental and JygCr-1KO cells were seeded into a 12-well dish in triplicate at $5x10^4$ cells/well and cultured for 24, 48 and 72 hrs. Cells were then harvested and counted using hemocytometer. ***In Vivo:*** JygMC(A) parental and JygCr-1KO cells were injected into both fourth mammary fat pads of 7 and 8 animals respectively and tumor growth was measured twice a week.

Supplementary References:

1. Pfaffl MW. A new mathematical model for relative quantification in real-time RT-PCR. Nucleic Acids Res. 2001; 29(9):e45.
2. Saeed AI, Bhagabati NK, Braisted JC, Liang W, Sharov V, Howe EA, Li J, Thiagarajan M, White JA and Quackenbush J. TM4 microarray software suite. Methods Enzymol. 2006; 411:134-193.
3. Rhodes DR, Kalyana-Sundaram S, Mahavisno V, Varambally R, Yu J, Briggs BB, Barrette TR, Anstet MJ, Kincead-Beal C, Kulkarni P, Varambally S, Ghosh D and

Chinnaiyan AM. Oncomine 3.0: genes, pathways, and networks in a collection of 18,000 cancer gene expression profiles. Neoplasia. 2007; 9(2):166-180.
4. Tabchy A, Valero V, Vidaurre T, Lluch A, Gomez H, Martin M, Qi Y, Barajas-Figueroa LJ, Souchon E, Coutant C, Doimi FD, Ibrahim NK, Gong Y, Hortobagyi GN, Hess KR, Symmans WF, et al. Evaluation of a 30-gene paclitaxel, fluorouracil, doxorubicin, and cyclophosphamide chemotherapy response predictor in a multicenter randomized trial in breast cancer. Clin Cancer Res. 2010; 16(21):5351-5361.
5. Hatzis C, Pusztai L, Valero V, Booser DJ, Esserman L, Lluch A, Vidaurre T, Holmes F, Souchon E, Wang H, Martin M, Cotrina J, Gomez H, Hubbard R, Chacon JI, Ferrer-Lozano J, et al. A genomic predictor of response and survival following taxane-anthracycline chemotherapy for invasive breast cancer. Jama. 2011; 305(18):1873-1881.
6. Gluck S, Ross JS, Royce M, McKenna EF, Jr., Perou CM, Avisar E and Wu L. TP53 genomics predict higher clinical and pathologic tumor response in operable early-stage breast cancer treated with docetaxel-capecitabine +/- trastuzumab. Breast Cancer Res Treat. 2012; 132(3):781-791.
7. Desmedt C, Piette F, Loi S, Wang Y, Lallemand F, Haibe-Kains B, Viale G, Delorenzi M, Zhang Y, d'Assignies MS, Bergh J, Lidereau R, Ellis P, Harris AL, Klijn JG, Foekens JA, et al. Strong time dependence of the 76-gene prognostic signature for node-negative breast cancer patients in the TRANSBIG multicenter independent validation series. Clin Cancer Res. 2007; 13(11):3207-3214.
8. Curtis C, Shah SP, Chin SF, Turashvili G, Rueda OM, Dunning MJ, Speed D, Lynch AG, Samarajiwa S, Yuan Y, Graf S, Ha G, Haffari G, Bashashati A, Russell R, McKinney S, et al. The genomic and transcriptomic architecture of 2,000 breast tumours reveals novel subgroups. Nature. 2012; 486(7403):346-352.
9. Radvanyi L, Singh-Sandhu D, Gallichan S, Lovitt C, Pedyczak A, Mallo G, Gish K, Kwok K, Hanna W, Zubovits J, Armes J, Venter D, Hakimi J, Shortreed J, Donovan M, Parrington M, et al. The gene associated with trichorhinophalangeal syndrome in humans is overexpressed in breast cancer. Proc Natl Acad Sci U S A. 2005; 102(31):11005-11010.
10. Sorlie T, Tibshirani R, Parker J, Hastie T, Marron JS, Nobel A, Deng S, Johnsen H, Pesich R, Geisler S, Demeter J, Perou CM, Lonning PE, Brown PO, Borresen-Dale AL and Botstein D. Repeated observation of breast tumor subtypes in independent gene expression data sets. Proc Natl Acad Sci U S A. 2003; 100(14):8418-8423.
11. Luistro L, He W, Smith M, Packman K, Vilenchik M, Carvajal D, Roberts J, Cai J, Berkofsky-Fessler W, Hilton H, Linn M, Flohr A, Jakob-Rotne R, Jacobsen H, Glenn K, Heimbrook D, et al. Preclinical profile of a potent gamma-secretase inhibitor targeting notch signaling with in vivo efficacy and pharmacodynamic properties. Cancer Res. 2009; 69(19):7672-7680.
12. Ran FA, Hsu PD, Lin CY, Gootenberg JS, Konermann S, Trevino AE, Scott DA, Inoue A, Matoba S, Zhang Y and Zhang F. Double nicking by RNA-guided CRISPR Cas9 for enhanced genome editing specificity. Cell. 2013; 154(6):1380-1389.

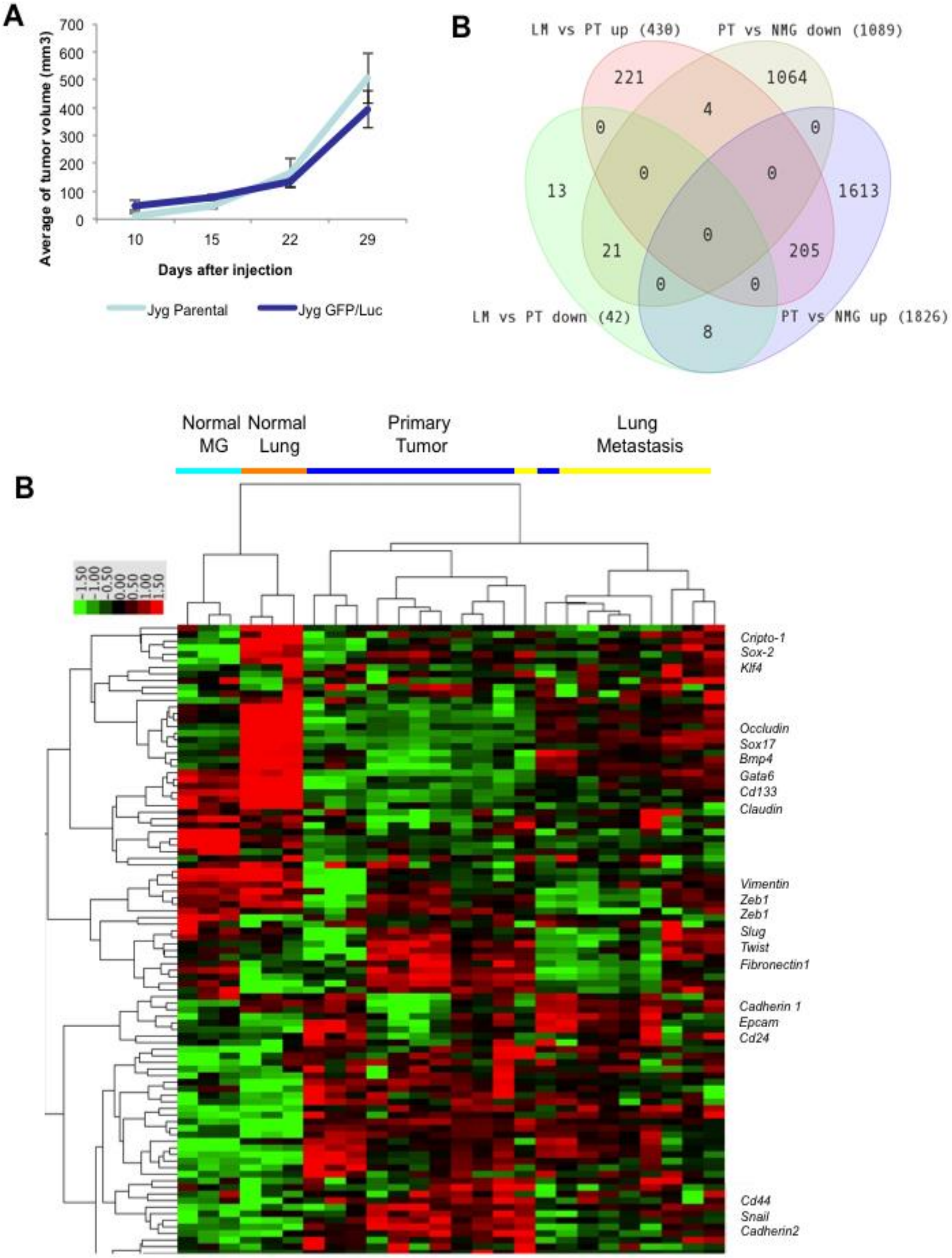
A
700
600
500
400
300
200
100
0
Average of tumor volume (mm3)
10
15
22
29
Days after injection
Jyg Parental
Jyg GFP/Luc
B
LM vs PT up (430)
PT vs NMG down (1089)
221
1064
4
0
0
13
0
0
1613
0
21
205
0
0
8
LM vs PT down (42)
PT vs NMG up (1826)
Normal MG
Normal Lung
Primary Tumor
Lung Metastasis
B
-1.50
-1.00
-0.50
0.00
0.50
1.00
1.50
Cripto-1
Sox-2
Klf4
Occludin
Sox17
Bmp4
Gata6
Cd133
Claudin
Vimentin
Zeb1
Zeb1
Slug
Twist
Fibronectin1
Cadherin 1
Epcam
Cd24
Cd44
Snail
Cadherin2

Figure S1. JygMC(A) mammary tumors and global gene expression in primary tumor and pulmonary metastasis. (A) Representation of mammary PT volume of parental line and tagged (GFP/Luc) cells (n=3 animals/group, ±SD). **(B)** Venn-diagram depicting the number of genes that were up- and down-regulated in PT vs. NMG and PT vs. LM. **(C)** Unsupervised clustering of the microarray data utilizing the same genes present on our customized NanoString nCounter® Gene Expression Codeset. PT: Primary Tumor; NMG: normal mammary gland; LM: Lung Metastasis.

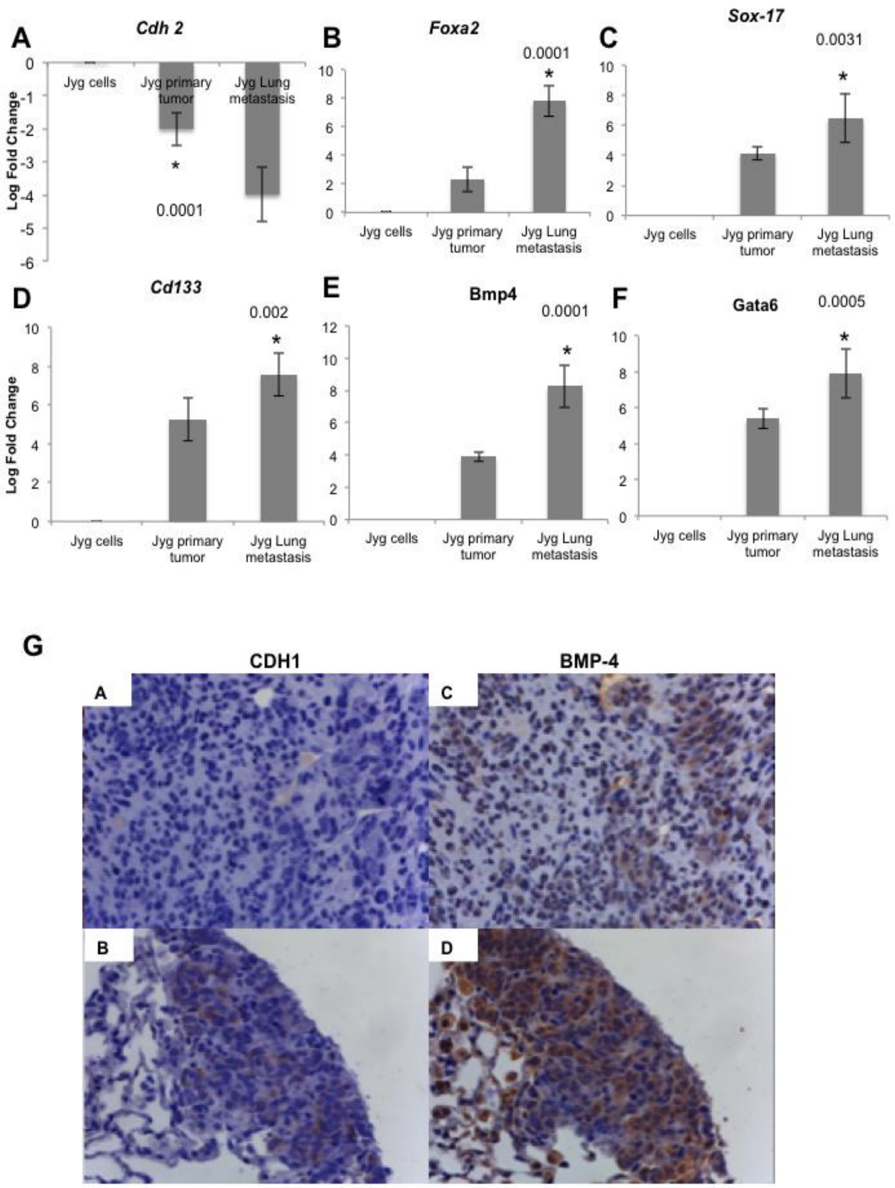

Figure S2. Gene and protein expression in primary tumor and lung metastasis. (A-F) Microarray versus NanoString qRT-PCR validation. Three

samples from different passages of JygMC(A)-GFP/Luc cells were used as a calibrator sample. *Hprt* was used as a normalizer. Seven matched-pair samples of JygMC(A) primary tumor and lung metastasis were used for qRT-PCR validation ±SD. **(G)** Immunohistological analysis of (A, C) Primary tumor and (B, D) Lung metastasis using antibodies directed against (A, B) CDH1; (C,D) BMP-4. Brown staining represents positivity.

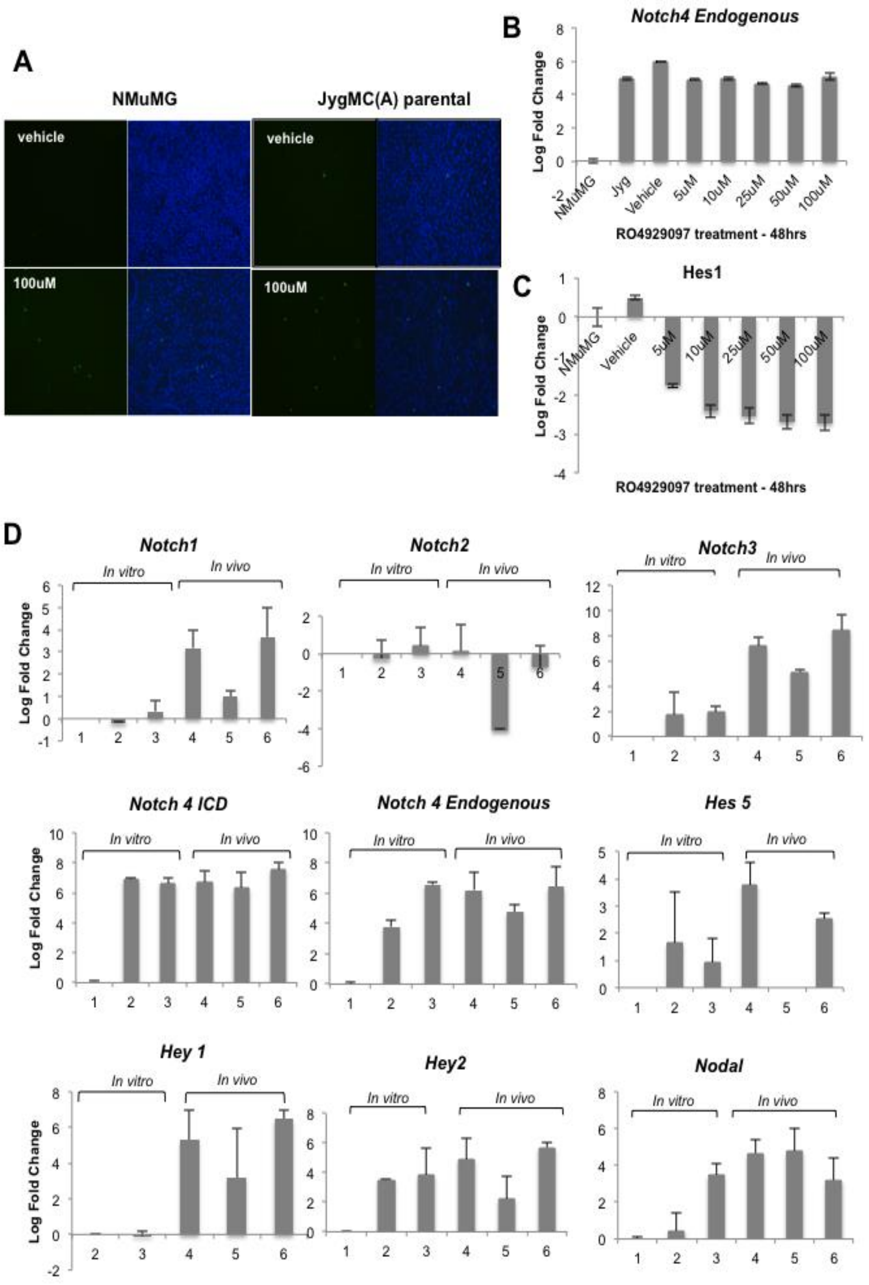

Figure S3. Gamma-secretase inhibitor treatment on JygMC(A) cells and gene expression of Notch signaling members, downstream effectors and Nodal (A) TUNEL assay after treatment with RO4929097 gamma-secretase

inhibitor. Field representation of NMuMG and JygMC(A) cells after 48hrs of treatment. **(B)** qRT-PCR for *Notch4* endogenous and **(C)** *Hes*1 in JygMC(A)-GFP/Luc cells treated with different concentrations of RO4929097 gamma-secretase inhibitor for 48hrs. **(C)** qRT-PCR of Notch signaling members, downstream effectors and related genes. *Notch 1-4;* downstream effectors, *Hey1*, *Hey2* and Hes5*;* TGF-β family member, *Nodal* in (1) NMuMG (calibrator), (2) JygMC(A) monolayer, (3) JygMC(A) spheres, (4) primary tumor, (5) lymph node and (6) lung metastasis. Data represents mean of 2-3 samples in duplicate, ±SD.

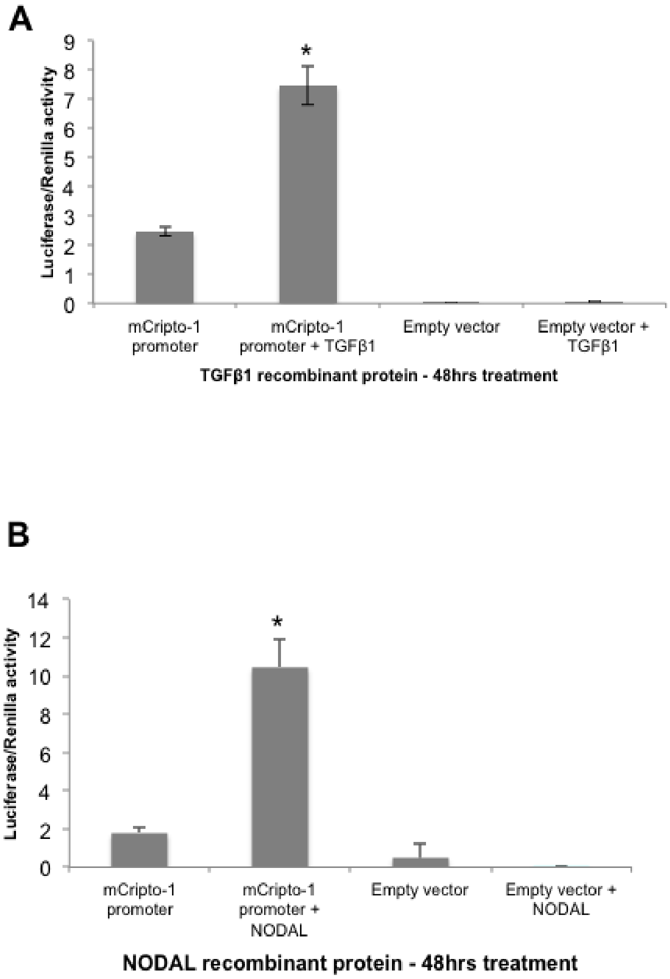

Figure S4. TGFβ1 and NODAL modulate *Cripto-1* promoter luciferase activity in F9 cells. Dual luciferase assay of transiently transfected F9 cells with *Cripto-1* promoter luciferase reporter vector. **(A)** Cells were treated with 10ng/mL of TGFβ1 recombinant protein for 48hrs. **(B)** Cells were treated with 100ng/mL of Nodal recombinant protein for 48hrs. Data are representative of duplicate samples ±SD, *P<0.01, as compared to untreated cells.

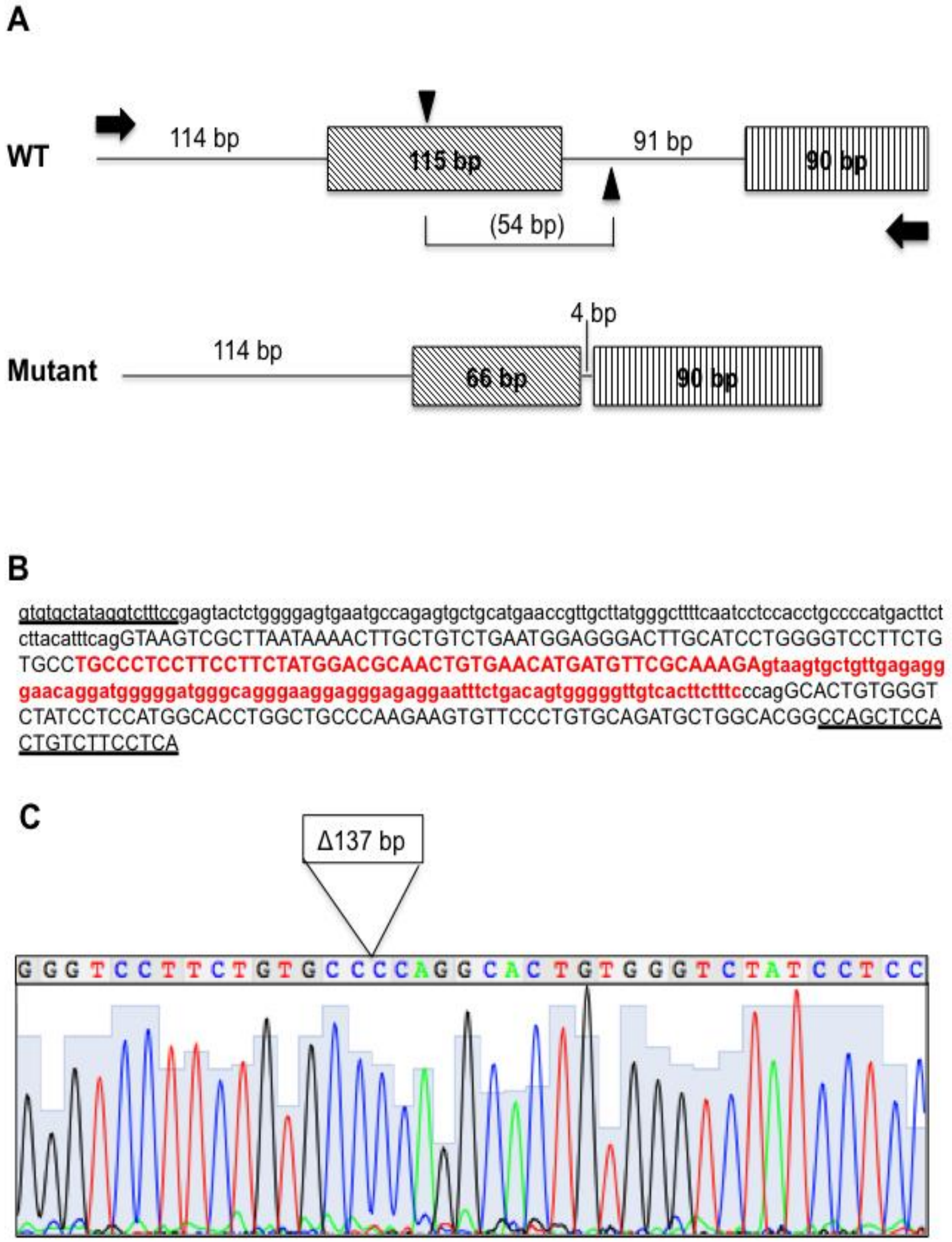

Figure S5: **Generation of JygMC(A) mutant cells using CRISPR-Cas9n. (A)** Schematic representation of wild type (WT) and mutant allele of mouse *Cripto-1*.

Thin lines and hatched boxes represent introns and exons respectively. Numbers represent the actual base pairs (bp) of introns and exons. Primers (5'-gtgtgctataggtctttcc-3' and 5'-tgaggaagacagtggagctgg-3') used to amplify the fragments for sequencing are marked by solid arrows. Solid triangles mark the position of nick generated by Cas9n. Distance between two nicks (54 bp) is marked. **(B)** Portion of *Cripto-1* DNA sequence showing deleted bases in bold red letters. Intron sequences are in small letters. **(C)** Sequence analysis of PCR product of mutant clone. Position of 137 bp deletion is marked.

Table S1: Primary mammary tumor and metastasis frequency of JygMC(A)-GFP/Luc cell line (Groups GA-GD) versus JygMC(A) parental line (Groups PA-PD).

Groups	Limiting Dilution (cells/gland)	Primary Tumor Frequency (%)	Lungs Metastasis Frequency (%)	Liver Metastasis Frequency (%)	Spleen Metastasis Frequency (%)	Kidney Metastasis Frequency (%)
GA	500,000	3/3 (100)	3/3 (100)	2/3 (66.67)	0/3 (0)	1/3 (33.34)
GB	50,000	3/3 (100)	3/3 (100)	2/3 (66.67)	0/3 (0)	0/3 (0)
GC	5,000	3/3 (100)	3/3 (100)	1/3 (33.34)	0/3 (0)	1/3 (33.34)
GD	500	2/3 (66.67)	2/3 (66.67)	0/3 (0)	0/3 (0)	0/3 (0)
PA	500,000	3/3 (100)	3/3 (100)	2/3 (66.67)	0/3 (0)	2/3 (66.67)
PB	50,000	3/3 (100)	2/3 (66.67)	1/3 (33.34)	1/3 (33.34)	1/3 (33.34)
PC	5,000	3/3 (100)	3/3 (100)	1/3 (33.34)	0/3 (0)	2/3 (66.67)
PD	500	2/3 (66.67)	2/3 (66.67)	0/3 (0)	0/3 (0)	0/3 (0)

Table S2: Oligonucleotides sequences used for quantitative real-time PCR

	Gene Symbol	Official Full Name	Primers sequences
Target Genes	Notch1	notch1	Forward: CAACATCTCTGATTGGTCCGAGG
			Reverse: GCATCCTGAAGCACTGGAAAGG
	Notch2	notch2	Forward: CCTGATTGGTTCTGGGACAAGTG
			Reverse: CATCTTCCGACAGCAAAGCCTC
	Notch3	notch3	Forward: GGATTTGAGGGGTGCTGAAGTG
			Reverse: CTGGGACAGAGAGAAAGGGATGAG
	Notch4/Int3	notch4	Forward: CTCCAGCTCATTCGGCGCCGGCGAC
			Reverse: GCCTGGGGGAGCTCTCCACAGCTGC
	Notch4/Full length	notch4	Forward: GCCAAGGTCAGGAACACAGA
			Reverse: CACCCAGTTCTGTCTCGCAT
	Hes1	hairy and enhancer of split 1	OriGene Technologies. Rockville, MD
	Hes5	hairy and enhancer of split 5	Forward: TTCCTTTGTATGGGTGGGTGC
			Reverse: GAAGCCTTCAGAACAGCCTGTG
	Hey1	hairy/enhancer-of-split related with YRPW motif 1	OriGene Technologies. Rockville, MD
	Hey2	hairy/enhancer-of-split related with YRPW motif 2	Forward: TGAAGATGCTCCAGGCTACAGG
			Reverse: TGCTGAGATGAGAGACAAGGCG
	Tdgf-1/ Cripto-1	teratocarcinoma-derived growth factor 1	Forward: GACTGGGGAAACAGAGTGGA
			Reverse: ACTCGGGTGTTTGGGTTTTC
	Nodal	nodal	Forward: TGCTGAAACGATACCAACCCC
			Reverse: CCTGCCATTGTCCACATAAAGC
	Cdh1	cadherin1	Forward: TGGATAGAGAAGCCATTGCCAAG
			Reverse: TTCCCCATTTGATGACACGG
	Cdh2	cadherin2	Forward: CTTGCTTCAGGCGTCTGTGGA
			Reverse: TCTCACAGCATACACCGTGC
	Bmp4	bone morphogenetic protein 4	Forward: TTCAAGATTGGCTCCCAAGAA
			Reverse: CCTAGCAGGACTTGGCATAATAA
	Foxa2	forkhead box A2	Forward: GAGACTTTGGGAGAGCTTTGAG
			Reverse: GATCACTGTGGCCCATCTATTT
	Gata6	GATA binding protein 6	Forward: GCGCCTCCTCTCTCCTTTTT
			Reverse: GCGCTACTCCAACCTGACTT
	Pecam1	platelet/endothelial cell adhesion molecule 1	Forward: GAAGTGTCCTCCCTTGAGCC
			Reverse: GCCTTCCGTTCTTAGGGTCG
	Sox17	SRY (sex determining region Y)-box 17	Forward: CGAGCCAAAGCGGAGTCTC
			Reverse: TGCCAAGGTCAACGCCTTC
	Prom1	prominin 1	SaBiosciences PPM04774A-200
Endogenous genes	Hprt	hypoxanthine phosphoribosyltransferase 1	Forward: CCTAAGATGAGCGCAAGTTGAA<br>Reverse: CCACAGGACTAGAACACCTGCTAA
	Gapdh	glyceraldehyde 3-phosphate dehydrogenase	Forward: CGTCCCGTAGACAAAATGGT<br>Reverse: TTGATGGCAACAATCTCCAC

Table S3: The nucleotide sequence of the mouse Cripto-1 promoter

The nucleotide sequence of the mouse *Cripto-1* promoter

2157bp from ATG start site

```
CCTTCAGTCTGCCACAGGCGGCTCATAGGTGAAGATTTCCAGAGTGGGTTGGCTCCGGGA
GACACTGGGAAGCTGAGAGTGCTGGAAGAGCAGGCTTAGACCTCCCATTGGGAACCAGGG
GGAGTGGGGTGAAAGACAAAAAAATAAGCAGTTTACAATCCAGTGTTAGTAAAGATCAAC
TCTGGGGTGGAGCAGTCGTTATCTCTCTGCGCTTTCCAGTTCAAGGCCACAGCTGGAAGC
CAGCTTTGGGAGGAAAGGTGCCCGGAAGGACTTTCCAGAGCAAAGGACCAAAGAAGCCAG
TTCTAGGGCACAGTCAGACCCTCCAGCTAGGGCTTAGGAAACTGAATACAGATACCAGTA
ACACAGCGAAACATGCAAATCCAAACAACCAAATACGAATTTCTCACATCAGGAGTCAGC
AGACGCCAAGCTACAGATGTTTGCTGCAGCCAGTGTGGACAAGTCCTGAGAGAGGGGCTC
AGTACACACGAAGGTTCAGGGGTGGGATTCTGGGAAAACATTGTGGAAGCATTCACAGAG
TAAGCAAGCAATTTCAGGGCTCAGTTATTTACCAGGACACACCCTCAAGTATCCCCAAGT
GATCATGGAAAGGCAGACACTGTAGAAGTGTGTCCCGCCAGGATAAAATAATTCCTTTAG
TCTACATTGGTAGAAAACATAAGCCACACAGAAACAAAAGGTGTCGCCCTCCTAATCCCT
TCTCCACTCCTCCAGTCTCAATGCAGGAATCTGGTCTTTCTGATCCCTACAGGGAACTAG
TGTATTCAGACTAGGGTTCTGTTTGTCAAGGTCTCAGTGAAATCAGCCATTAGCTTGTTA
ATGAAGGAGGCCCAGCCTTCTCGGAAATAGCAGATTCCCCCCCCCACCACCACCACCAA
TTGCCCCTCACCCCCTAGGGTTTCAGGAACTGGAAAAGTGGCTGGAAACTCAGAGTGACA
GGGGATGGGGGTGGGGGGGAGTAGAGAGACTGGGCGTGTACAGAATTCCCAGGCCTGTCG
GGGTCTTCACTCTCGACTAAAACGGAAGTAAATCACCATAGAAGCCAGGGGCGAAGGTGA
GGGAGGGTTTTGTGTTTTGTCATTTTGATCTTGGGTCTTAGCTGTGCTTGGTGATACGTG
CTTGTAATCTCGGAATTTGGTGAAACGGCAGCACAAAGGTCGCCGAGTTTCACGCCAGCC
TGATCTTGAGACCCTCAAAAGAAAGCAAGGAAGAAAGGAAAAGTGGTCTTGCCCTGTAAG
CGCCGGCTGGCTTTAAACTCACTGAAGGCCAGGCTGACCTCTAACTCACTTCAATCCTCC
GATCTCCACCTCCCCAAGTCCTAGAACTACGGGCCTTTGCCACCAGGTCCAGCTTTTGTG
ATCTATATAATAATGTGAGATATATATCAAAATGTATCACTCAGGGATGTGTTTCTCAGC
GGGCTCAGCCACAGACGAGCACCTAGCAACAGAGAGGTACAACCCCACTCGCAAGTCAAG
GCCCTCCCCATCCGCCCACAAGAAACCGAAGCCAGTAACCCGCAGCTTAGCACTGAGTTG
AGGACCCGGAAGAATCAAGTGACTTCCAATAGTGCTTGTGAGGCCACTGATTTTTAAGCT
TCAGGCAGCCGCTCGCCCCAGCGCCCACCCGCAGGCTCTGGGCCAGGGCTTGCAATTCAG
ACGCTGCAGGCAAAACCTCTAGCCCAAACCCAGACTCAAATCCCTCCCGACATTGACCTT
CCCCCACACCCAAACAAGTCCTCGCCAGCCGTCAGCGCATCTTCAAGGCCTCCAGCACCC
GGCTCTACTGGGAGTAACGACGAACGCAAATGCAGAGTTAAAATTTGGCAGTTACCCTGG
AGGTGGTGGAGCACGCCTTTAATCCCTTAGGGGCAGGCAGAAGATCTCTTGGGTTCGAGG
CCAGCCTGGTCTACCTAGGGAAACTATTCTTGAAAAACAAACAAAACAACAAATTTTGAT
AATGAAGTTTCAAATGCCTGTATGGTGTTTAGACTTTCCTAATTACTTTCGTGTTTGAAC
ACCTTGGTAGAATAACTAAACTCTTCAACTCCCTTTACCTATAGAAAATTTACATATAAC
CAGACTATAGAGAATTTACATAAAAACCAAACTAGTTCACTTCCCTCGGAGCTTCCTGGC
CTAGTAATGGGCGTGGGGCCCCGTAATGGGCGTGGGGCCCCGTCTCCCCGCCCACCTGAG
GGTCCTACTCAACACCTTAAGTTTCTTTTAAGGGAGGTATATTCCTCCGAAGTCCTCAAT
CACAAACCGTTTATAGGAAGAAGAGGCCCCATCCCCTGCCGGTCTACACGGAGATCTTGG
CTGCTAACTTCCCACAGACTCTCCAGGACGGGGGCCTCTCTCATTTGGCATATCTTTCTT
TTTAATCTACTGTTTTCATTTTGTGAAATTAGCCTTTGGGTGTTTCGAGAATGGCTTTAT
GAACTAAAGCCATCTGCTAATATTGTGTTTCTTGTCTTTTCCTCCAACGTTTTTACGAGC
CGTCGAAGA
```

Datasets

Dataset S1: NanoString mRNA data

Dataset S1A: The targeted region and corresponding sequences of embryonic stem cell and EMT-MET markers selected for a custom NanoString probe set.

Dataset S1B: 31 genes differentially expressed between primary tumor carcinoma vs. primary tumor EMT - t-Test p-value <0.05.

Dataset S1C: 38 genes differentially expressed between primary tumor carcinoma vs. lung Metastasis - t-Test p-value <0.05.

Dataset S1D: 65 genes differentially expressed between primary tumor EMT vs. lung Metastasis - t-Test p-value <0.05.

Dataset S1E: 88 genes differentially expressed among all samples - ANOVA test p-value <0.01.

Dataset S2: Microarray mRNA data

Dataset S2A: 2915 differentially expressed genes (Primary Tumor vs. Normal Mammary Gland).

Dataset S2B: 472 differentially expressed genes (Lung Metastasis vs. Primary Tumor).

Dataset S2C: Top KEGG pathways enriched in primary mammary tumors vs. normal mammary gland.

Dataset S2D: Top KEGG pathways enriched in lung metastasis vs. primary tumors.

Dataset S3: Nanostring versus Microarray

Dataset S3A: Summary of the microarray gene expression profiling using the nanostring gene list.

Dataset S3B: Seven common genes between global microarray and nanostring gene expression profile.

Dataset S4: Oncomine data

Dataset S4A: Differentially expressed genes between the JygMC(A) primary mammary tumors vs. normal mammary gland and human breast cancer subtypes datasets.

Dataset S4B: Differentially expresssed genes between metastasis vs primary tumor datasets.

Dataset S5: Nanostring miRNA data

Dataset S5A: Top miRNAs found on malignant and normal mammary gland, lung metastasis and JygMC(A) cell line.

Dataset S5B: miRNAs modulators of the common six validated genes between microarray and nanostring
